# Task-sharing to promote caregiver mental health, positive parenting practices, and violence prevention in vulnerable families in Sierra Leone: a pilot feasibility study

**DOI:** 10.1186/s12888-024-06209-w

**Published:** 2024-11-11

**Authors:** Alethea Desrosiers, Indrani Saran, Ariana M. Albanese, Cara M. Antonaccio, Sarah E. Neville, Rebecca Esliker, Musu Jambai, Mahmoud Feika, Theresa S. Betancourt

**Affiliations:** 1 Department of Psychiatry and Human Behavior, Brown University, Warren Alport Medical School, 345 Blackstone Blvd, Providence, RI 02906 USA; 2 Boston College School of Social Work, 140 Commonwealth Avenue, Chestnut Hill, MA 02496 USA; 3 University of Makeni, VWQV+54G, Lunsar-Makeni Highway, Makeni, Sierra Leone; 4 Caritas-Freetown, Savage Road, Freetown, Sierra Leone

**Keywords:** Task-sharing, Caregiver mental health, Parenting practices, Early childhood development, Violence prevention

## Abstract

**Background:**

The prevalence of household violence in low- and middle-income countries (LMICs) is high, and exposure to violence has significant adverse effects on both mental health and child development across generations. Evidence-based services to improve parenting practices and reduce household violence in LMICs are scarce, particularly across rural regions of West Africa. This study explored the feasibility, acceptability, and potential benefits of an evidence-based home-visiting intervention to promote early childhood development and reduce household violence—the Family Strengthening Intervention for Early Childhood Development and Violence-Prevention (FSI-ECD + VP)—among vulnerable families in rural regions of Sierra Leone.

**Methods:**

Eighty dual-caregiver households in the Makeni region of Sierra Leone were included in the study (*N* = 160 caregivers; 73% female). Eligibility criteria included having at least one child aged 6–36 months and elevated scores (> 62.5) on the Difficulties in Emotion Regulation Scale (DERS). Community Health Workers (CHWs) employed in the Makeni region completed a 3-week FSI-ECD + VP training. Families were randomized to receive either the FSI-ECD + VP or treatment as usual (TAU). Research assistants blinded to treatment assignment assessed caregiver mental health, caregiver-child interactions, and household violence at baseline, post-intervention, and 3-month follow-up time points.

**Results:**

Triangulation of quantitative and qualitative data showed that caregivers, CHWs, and supervisors generally perceived the intervention as beneficial, feasible, and acceptable. Mixed effects models showed that caregivers who received the FSI-ECD + VP had significantly improved caregiver-child relationship outcomes compared to TAU as assessed by the Home Observation for Measurement of the Environment and the Observation of Caregiver-Child Interactions at post-intervention. Preliminary data also suggests that caregivers receiving the FSI-ECD + VP were less likely to have experienced intimate partner physical violence during the post-intervention period, and had lower symptoms of anxiety and depression at 3-month follow-up.

**Conclusions:**

FSI-ECD + VP delivery by CHWs in Sierra Leone may be feasible and acceptable; it may also help improve caregiver-child interactions and reduce the likelihood of household violence among vulnerable families with young children. Task-sharing approaches may help increase acceptability and access to evidence-based behavioral interventions that promote early childhood development and violence prevention among families in rural regions of Sierra Leone and other similar settings.

**Trial registration:**

The study is registered in clinicaltrials.gov (NCT 03045640; 07/22/2020). This study follows the Consort 2010 guidelines for reporting of clinical trials.

**Supplementary Information:**

The online version contains supplementary material available at 10.1186/s12888-024-06209-w.

## Introduction

Exposure to war, trauma, and other humanitarian crises can have persistent, intergenerational mental health effects. The World Health Organization estimates that 75% of children in low- and middle-income countries (LMICs) experience some form of violent or psychologically damaging discipline at home. Experiencing or witnessing household violence during early childhood increases risks for emotion regulation and other psychological problems—including post-traumatic stress disorder, externalizing and internalizing behavioral difficulties, and school problems [1, 2]. Additionally, caregiver mental health problems, particularly maternal depression—which is exacerbated in the context of intimate partner violence (IPV)-- are associated with serious developmental problems in infants, children, and adolescents [3, 4]. It is also associated with higher rates of malnutrition and stunting, lower birth weight, and lower completion of infant immunization schedules [5]. Although maternal depression is more common among women in LMICs than high-income countries, it is often untreated [6], and very few programs consider the contributions of both male and female caregivers to overall family functioning and nonviolent interactions in the home.

Sierra Leone is a West African country that has been affected by violence and adversity over the last several decades (i.e., civil unrest, outbreaks of the Ebola virus and other diseases, and climate-change related disasters). Research on the intergenerational impact of the 11-year civil war has shown that exposure to violence is related to poor parent/caregiver mental health and harsh parenting practices, which adversely affect child development [7–11]. The 2017 Sierra Leone Multiple Indicator Cluster Survey found that 85% of children aged 3–4 and 67% of those aged 1–2 experience violent discipline [12]. Given that trauma exposure is related to poor caregiver emotion regulation, household violence, mental health and poor child development outcomes, evidence-based interventions focused on enhancing caregiver-child interactions (including father/male caregiver involvement), improving caregiver emotion regulation and caregiver mental health, and promoting alternatives to harsh discipline practices are urgently needed [5, 6].

**Fig. 1:**
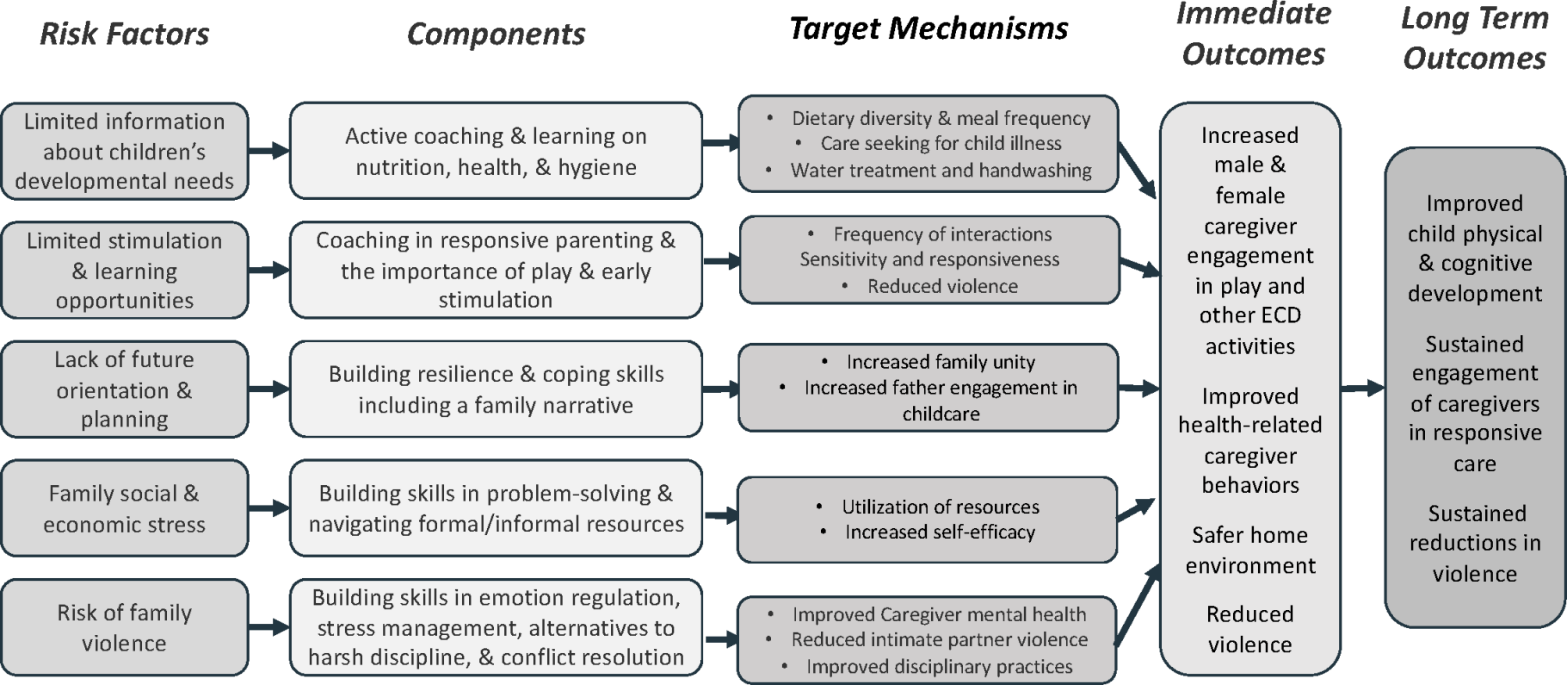
FSI-ECD + VP theory of change

In prior research with families experiencing extreme poverty in post-genocide Rwanda, we developed and evaluated the Family Strengthening Intervention for Early Childhood Development and Violence Prevention (FSI-ECD + VP), a home-visiting intervention that is delivered by nonspecialists and engages both male and female caregivers across a range of family configurations [13, 14]. The FSI-ECD + VP targets caregiver emotion regulation, male caregiver engagement and caregiver-child interactions as major mechanisms to prevent the intergenerational transmission of emotional and behavioral difficulties related to past trauma (See Fig. 1) among families with young children (i.e. at least one child < 36 months of age). It has demonstrated effectiveness in improving caregiver mental health, reducing IPV and harsh discipline practices, and promoting healthy child development [9, 12]. The FSI-ECD + VP is a promising approach for targeting key risk factors linked to poor child outcomes [7, 8]. Critical for LMICs and similar settings, the FSI-ECD + VP can be delivered by nonspecialist health workers with strong supervision and performance monitoring; thus, it has the potential to address the urgent need for evidence-based behavioral interventions among vulnerable families in resource constrained settings like Sierra Leone.

Current leadership in the Government of Sierra Leone is focused on implementing new mental health initiatives to respond to the impact of the country’s history of compound adversity. This includes investing in their national community health worker program to address healthcare workforce limitations that impede delivery of evidence-based behavioral interventions to vulnerable families. In 2017, the Sierra Leone Ministry of Health and Sanitation launched the *National Community Health Worker Policy*, which defines a key role for community health workers (CHWs) in the delivery of preventative maternal and child health services and linking communities with the health system. Coupled with the Government of Sierra Leone’s investments and policies, extant literature supports the feasibility, acceptability and effectiveness of using task-sharing approaches (i.e., CHWs and nurses) to increase the availability of evidence-based behavioral interventions for underserved populations throughout Sub-Saharan Africa [15]. Building CHW capacity to deliver evidence-based family home visiting interventions like the FSI-ECD + VP in Sierra Leone could help address the lack of evidence-based services available to promote positive parenting practices, caregiver mental health and emotion regulation, male engagement, and nurturing caregiver-child interactions [16].

This pilot feasibility study evaluated a culturally adapted version of the FSI-ECD + VP delivered by CHWs in rural regions of Sierra Leone to vulnerable families with children aged 6–36 months. We used a mixed methods approach to assess the feasibility and acceptability of the intervention, as well as preliminary effects of the FSI-ECD + VP on caregiver mental health, emotion regulation, and caregiver-child interactions, compared to control families who received standard care. We hypothesized that the FSI-ECD + VP would be feasible, acceptable, and appropriate among caregivers, CHWs, and supervisors. We also hypothesized that caregivers who received the FSI-ECD + VP would show greater improvements in caregiver-child interactions and caregiver mental health and greater reductions in household violence compared with caregivers who received standard services.

## Methods

### Study design, setting and participants

We conducted a randomized controlled pilot feasibility study to evaluate the feasibility, acceptability, and preliminary effects of the culturally adapted FSI-ECD + VP on caregiver mental health, caregiver-child interactions, and household violence reduction among vulnerable families in the Makeni region of Sierra Leone. Families (*N* = 80) with children aged 6–36 months were randomized to receive CHW delivery of either the FSI-ECD + VP (*N* = 40 families) or standard maternal and child health home visiting services (*N* = 40 families).

Research assistants (RAs) collecting data and data analysts were blinded to participant assignment. Blinding of study participants was not possible due to the nature of the comparison and intervention conditions (e.g., CHWs received training in the FSI-ECD + VP and therefore were not blinded).

Makeni is the largest city and the provincial headquarter town in the Northern Province of Sierra Leone. It consists of both rural and urban regions. The most common forms of employment are agriculture and trade. The total population is 124,634 people.

We recruited and enrolled families from two rural districts in Makeni (Yoni and Binkolo) to participate in the pilot trial in July 2021. Inclusion criteria for families were as follows: (a) a Sierra Leonean household with cohabitating caregivers (e.g., father/mother, mother/grandmother, mother/partner) and child (aged 6–36 months) in which both caregivers aged 18 or older and (b) one caregiver scoring at least 62.5 on the Difficulties in Emotion Regulation Scale (DERS). The DERS cut-off score has been used successfully as a risk assessment screening tool in our prior studies in Sierra Leone [17], and difficulties with emotion regulation have been associated with an array of mental health problems as well as with interpersonal violence and poor parenting skills [5, 6]. For enrolled families with more than one child aged 6–36 months, all eligible children were included as study participants. We excluded families who did not meet all inclusion criteria and/or who were experiencing current suicidality, psychosis, or a serious medical condition as assessed by a study social worker in the local language (Mende or Krio, the latter being the most common language of Sierra Leone).

Families were recruited in coordination with the CHW Focal Person, who is the Ministry of Health and Sanitation Community Health Worker Program official responsible for coordinating the work of CHWs and supervisors within peripheral health units. Peripheral Health Units (PHUs) are the primary community-based health clinics within the Sierra Leone healthcare system. PHUs maintain records of families in the community who have sought services, thus we worked with the CHW focal person to identify families with a child aged 6–36 months through reviewing these records.

We recruited CHWs (*N* = 8) and supervisors (*N* = 2) from two PHUs in Yoni and Binkolo districts to deliver the FSI-ECD + VP and provide weekly supervision, respectively. In Sierra Leone, CHWs are selected by their community and trained to provide basic health services and health education. Guidelines for selection include gender parity, the ability to communicate, an interest in health, the ability to perform CHW tasks, the ability to read and write in the local language, aged 18 years or above, and physical and mental fitness to provide services. Inclusion criteria for CHWs were (a) assigned to the Peripheral Health Unit that provides health services in one of the two communities and (b) aged 18 years or older. Inclusion criteria for supervisors were (a) currently overseeing CHWs providing maternal and child health services in one of the two communities and (b) aged 18 or older. We excluded CHWs and supervisors who did not meet inclusion criteria. All participants provided oral consent prior to study participation. All study procedures were approved by the Boston College IRB (reference number 21.006.01) and the Sierra Leone Ethics and Scientific Review Committee. The study is registered in clinicaltrials.gov (NCT 03045640; 07/22/2020). Trial reporting follows the Standard Protocol Items: Recommendations for Interventional Trials (SPIRIT) guidelines.

### Procedures

#### Family strengthening intervention for early child development (FSI-ECD + VP)

The FSI-ECD + VP is comprised of several core components: (a) developing problem-solving, stress management, and emotion regulation skills; (b) cultivating positive parenting skills and fostering father/male caregiver engagement; (c) developing communication and conflict resolution skills; (d) exploring alternatives to harsh punishment and practicing non-violent child discipline.; (e) providing psychoeducation on nutrition and hygiene; and (f) promoting early stimulation and language learning. The FSI-ECD + VP integrates key elements of the evidence-based Family Based Prevention Intervention [25] and was culturally adapted to the Rwandan context through extensive community based participatory research methods involving Rwandan community advisory boards. The FSI-ECD + VP contains 12 modules delivered in the home via CHW “active coaching” in playful parenting and stimulation appropriate to the age and developmental level of the target child. Sessions are delivered once per week and last approximately 90 min, with both caregivers present for sessions.

Prior to launching this trial, the FSI-ECD + VP was translated into Krio and culturally adapted to the Sierra Leonean context using community-based research methods. This included meetings with community advisory board members in Makeni, local ECD experts in Sierra Leone, and Ministry of Basic and Senior Secondary Education Officials to review the intervention manual and provide feedback on culturally appropriate modifications. The “core content” of the intervention was not modified. The translated manual was also reviewed by the local Co-Investigator to check the accuracy of translated material. Adaptations were tracked using the FRAME-IS and reported in prior work [18, 19].

#### Standard services

Standard CHW care involves three educational sessions delivered to families in their homes following childbirth. Topics of home visiting sessions include skilled postnatal care for mothers, early initiation of breastfeeding and exclusive breastfeeding practices, adequate nutrition, immunization services and timely use of these services, hand washing and hygiene practices (including waste disposal and food hygiene) and building the capacity of family members to appropriately care for children under age 5. CHWs also conduct screenings for acute malnutrition and growth monitoring to identify early referrals, and they can provide family planning methods, deworming tablets and vitamins for acute malnutrition, and anti-malaria treatment. Each home-visiting session lasts approximately 60 minutes.

#### Training

FSI-ECD + VP training for CHWs and supervisors was held five days per week over the course of three weeks. The Sierra Leone-based Program Manager completed three weeks of training led by an FSI-ECD + VP expert in Rwanda to review intervention components and video tapes of session role plays and practice. The Program Manager then led an FSI-ECD + VP training with CHWs and supervisors, in consultation with the Rwandan FSI-ECD + VP expert. Training included didactic components, role plays, and feedback sessions to review strengths and weaknesses during role play and practice. CHWs and supervisors completed an FSI-ECD + VP competency assessment before and after training to ensure > 70% competency was achieved.

### Data collection

**Fig. 2:**
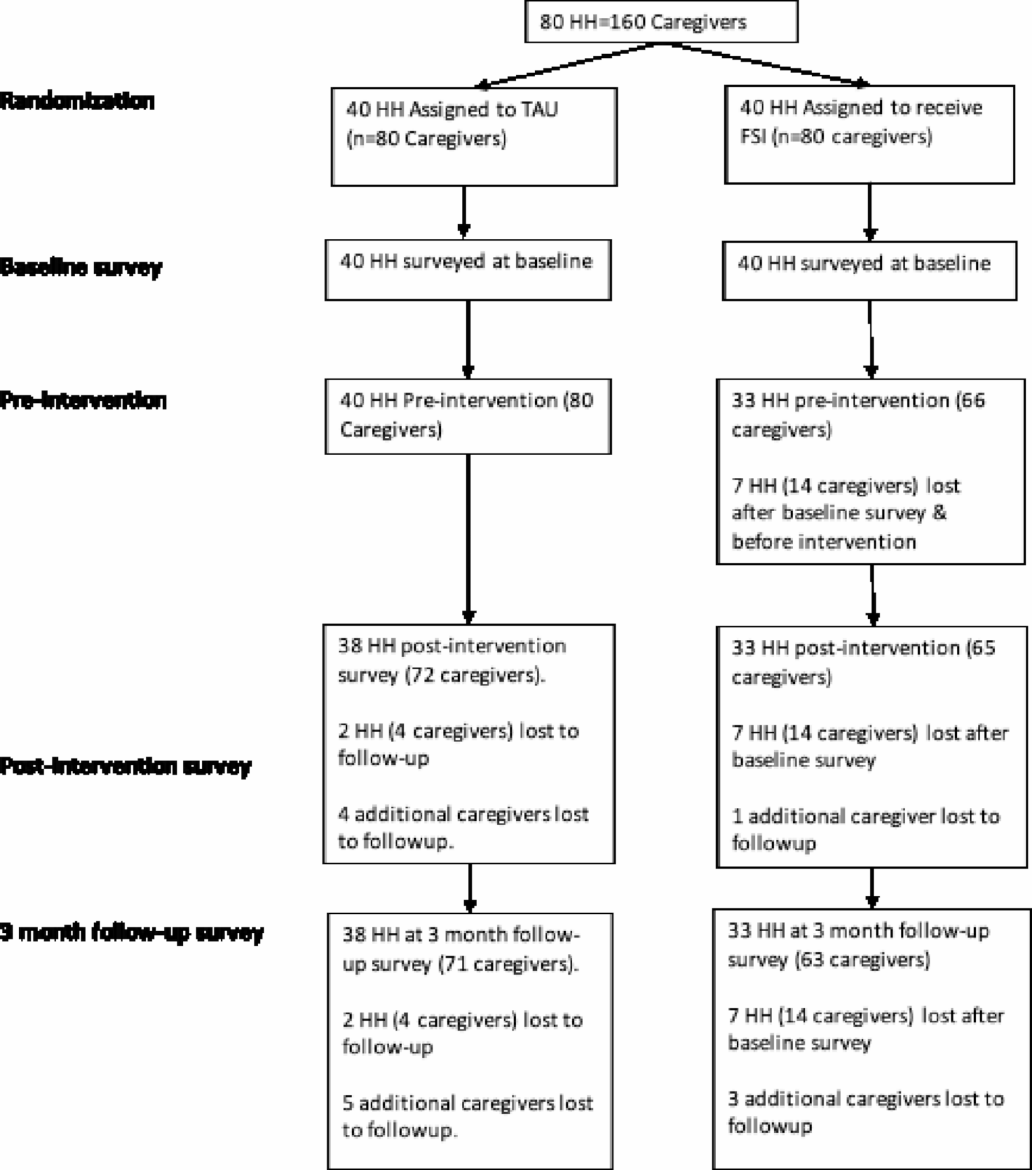
Consort diagram for the study

Families were randomized to receive the FSI-ECD + VP or standard services using the randomization allocation sequence in RedCap [20]. Trained RAs collected quantitative data on caregiver mental health, caregiver-child interactions, the home environment, and family functioning at baseline (August-October 2021), post-intervention (July-November 2022), and 3-month follow-up (December 2022-January 2023) (See Fig. 2 for consort diagram). Data was collected from both caregivers. A 6-month gap took place between baseline data collection and the start of the intervention. This was due to the timing of a mandatory training held by the Sierra Leone Ministry of Health and Sanitation that all CHWs were required to complete to maintain their status as CHWs. FSI-ECD session roll out occurred from March-September 2022. Post-intervention data collection started immediately after intervention completion, and 3-month follow-up started 3-months after post-intervention.

All quantitative measures were forward and backward translated into Krio following the WHO process of translation and adaptation of instruments and demonstrated strong reliability. RAs also collected quantitative and qualitative data on FSI-ECD + VP delivery from a randomly selected subset of caregivers (*N* = 8; 4 males and 4 females) and CHWs (*n* = 4), as well as the two CHW supervisors. A semi-structured interview guide was created for each participant type to obtain feedback on experiences with the intervention, including perceived benefits and perceptions of the feasibility, acceptability, and appropriateness of the FSI-ECD + VP. Study data was collected via password protected, encrypted tablets and de-identified and uploaded to Box, a secure server, HIPAA-compliant, cloud-based storage system.

### Measures

Scales previously validated for use in Sierra Leone, Liberia, and other conflict-affected countries in sub-Saharan Africa were used to assess caregiver mental health. Depression and anxiety symptoms were assessed using a Krio adaptation of the Hopkins Symptom Checklist-25 (HSCL-25; adapted for use in Sierra Leone), scored on past week symptom intensity (1 = not at all, 2 = a little, 3 = quite a bit, 4 = extremely) [21, 22]. Post-traumatic stress symptoms were assessed using a 16-item adaptation (0 = no, 1 = yes) of the Civilian Post-traumatic Stress Disorder Checklist used in Liberia [23, 24]. Caregiver emotion regulation was measured using the 36-item Difficulties in Emotion Regulation Scale (DERS; sum of 36 items [scored 1–5] with higher scores representing higher levels of dysregulation) [25]. Functioning was measured using the 12-item World Health Organization Disability Assessment Schedule (WHODAS) [26]. Caregiver-child relationships were assessed using the Home Observation for Measurement of the Environment and the Observation of Mother-Child Interaction [27–29]. The HOME is a 43-item adaptation of the infant/toddler Home Observation for Measurement of the Environment (HOME) Inventory that has been used previously in West Africa. In most cases we used the HOME reports from the primary caregiver. In 12 households where this was not available, we used the secondary caregiver’s report. The OMCI is a five-minute mother-child interaction observation that is scored along 19 items using published criteria. Because we assessed both male and female caregivers with the measure, we refer to it as the Observation of Caregiver-Child Interactions (OCCI). Experiences of IPV were based on questions from the Demographic and Health Surveys about experiences of physical and sexual violence with a partner in the past 12 months [30].

Feasibility, acceptability, and appropriateness were measured using an instrument developed by the Applied Mental Health Research group at Johns Hopkins University [31, 32]. Items were rated on a four-point scale in terms of agreement from 1 (“Not at all”) to 4 (“A lot”). This measure has been translated and validated in our prior work in Sierra Leone [17].

### Data analysis approach

This study uses a convergent parallel mixed methods design to integrate quantitative and qualitative evidence at the analysis stage, for the purpose of convergence [33]. Both sets of data were compared (triangulated) to examine if they reached the same conclusions about the impact of the FSI-ECD + VP intervention on families as well as its feasibility and acceptability.

### Quantitative data analysis

All quantitative analyses were conducted in Stata 15 [34]. Assuming a standard alpha level of 0.05, 80 families with two eligible respondents per family on average, and two time points, with assumptions of moderate intra-class (within-family) correlation (approximately 0.5), this pilot RCT had power of 0.80 to detect a standardized “medium” effect size of approximately 0.50 [35].

Seven intervention families withdrew from the study after randomization and the baseline survey but before receiving the intervention (Fig. 1). They were replaced by six families who were randomly selected from a replacement list of eligible families. The replacement families did not complete the baseline survey due to time and resource constraints, but they participated in the intervention and completed the post-intervention and follow-up assessments. We conducted an intent-to-treat (ITT) analysis that excluded these six intervention households since they were not part of the original randomization. However, we also conducted a robustness check that included these 6 replacement households. Of our originally randomized sample of 160 caregivers, 23 did not complete the post-intervention survey (14%) and an additional 3 caregivers did not complete the 3-month follow-up survey (for an overall loss to follow-up rate of 16%).

We used linear mixed models with restricted maximum likelihood estimation and unstructured covariances to assess the effects of the intervention at each follow-up time point. For most outcome measures, we included observations from both caregivers in the household; thus, we included random effects at the caregiver level (to account for clustering over time) and at the household level (to account for clustering within families). Since the HOME measure consisted of one observation per family, analyses with this outcome included only the household-level random effect. For binary outcomes, we used mixed effects logistic models.

Our models used dummy variables to indicate the post-treatment period and for the 3-month follow-up period, as well as intervention by time interactions to assess the effect of the intervention at each follow-up time period. The models adjusted for the caregiver’s age, gender, marital status, highest household education level, number of children under age 17, the focal child’s age (in months), focal child’s sex, and district (Binkolo vs. Yoni) (all measured at baseline). Given the 6-month delay between baseline data collection and FSI-ECD + VP delivery, we present the model-predicted differences in outcomes between the intervention and control groups at post-intervention and 3-month follow-up (using the “margins” command in STATA).

### Missing data

Since there were relatively little missing outcome data (< 10% of caregivers at each time point) and moderate loss-to-follow-up (14–16%), we conducted a complete case analysis. However, as a robustness check, we also conducted an analysis using multiple imputation by chained equations specifying linear regressions and using twenty imputed datasets. We performed the multiple imputation both excluding the 6 replacement households (i.e. an ITT) and also with them included. Auxiliary variables in the multiple imputation included child sex, caregiver gender, caregiver marital status, highest household education level, the number of children under 17 in the household, district, and intervention status (all measured at baseline). Some outcomes could not be included in the multiple imputation models because of convergence problems. In particular, the anxiety and depression subscales of the HSCL were highly correlated with the overall HSCL measure, and experiences of intimate partner violence in the past 12 months were correlated at the follow-up time periods (and were also relatively rare).

### Qualitative data analysis

Two study team members with qualitative methods experience used a rapid data condensation approach to analyze the qualitative data [36, 37]. First, they created a summary template for each interview based upon the broad domains of the semi-structured interview guide. As there were separate interview guides for caregivers, CHWs, and supervisors, three templates were created. After “testing” the summary template on one interview, templates were finalized, and each interview was double coded. The two coders met weekly to review the summaries and create a final summary for each interview through a discussion and consensus process. Final individual interview summaries were consolidated into matrices by participant type (caregiver, CHW, or supervisor), in which each row of the matrix represented a participant, and each column contained a summary of that participant’s response. Participant responses were analyzed using a thematic framework analysis approach in which a “memoing” approach was used to summarize the data within each column and inductively identify themes [24].

## Results

Descriptive statistics for the analysis sample of the original 80 households are displayed in Table 1 (baseline levels of outcomes for the sample are in Appendix Table A1). The primary caregiver was most often the mother (95%), whereas the secondary caregivers were usually fathers (50%) or grandmothers (30%). The highest level of education in the household was most frequently senior secondary education (53%). Approximately 5 children (SD = 3.2) under the age of 17 lived in the household. With regards to the focal child, 34% were female, and they were 21 months old on average (SD = 9.5). Caregivers were mostly female (73%) and married/in a relationship (76%), and they were 32 years old on average (SD = 12). Descriptive statistics comparing intervention households that remained in the study (*N* = 33) with households that left the study prior to receiving the intervention (*N* = 7) are included in Appendix Table A2.

**Table 1:** Sample descriptive statistics

	Control (*N* = 40 HH, 80 Caregivers)	Intervention (*N* = 40 HH, 80 Caregivers)	Full sample (*N* = 80 HH, 160 Caregivers)
	N (%) or Mean (SD)		
**Household Characteristics**			
District			
Yoni	17 (42.5%)	18 (45.0%)	35 (43.8%)
Binkolo	23 (57.5%)	22 (55.0%)	45 (56.2%)
Number of children < 17 yrs	5.15 (3.31)	5.00 (3.05)	5.08 (3.16)
Highest level of education in household			
Primary school	1 (2.5%)	5 (12.5%)	6 (7.5%)
Junior secondary	8 (20.0%)	8 (20.0%)	16 (20.0%)
Senior secondary	22 (55.0%)	20 (50.0%)	42 (52.5%)
Post-secondary	9 (22.5%)	7 (17.5%)	16 (20.0%)
Focal child age (months)	22.42 (9.73)	20.13 (9.29)	21.27 (9.52)
Focal child female	14 (35.0%)	13 (32.5%)	27 (33.8%)
Primary caregiver relationship with child			
Mother	38 (95.0%)	38 (95.0%)	76 (95.0%)
Grandmother	2 (5.0%)	1 (2.5%)	3 (3.8%)
Other	0 (0.0%)	1 (2.5%)	1 (1.2%)
Secondary caregiver relationship with child			
Father	19 (47.5%)	21 (52.5%)	40 (50.0%)
Grandmother	13 (32.5%)	11 (27.5%)	24 (30.0%)
Aunt	3 (7.5%)	3 (7.5%)	6 (7.5%)
Other	5 (12.5%)	5 (12.5%)	10 (12.5%)
**Caregiver Characteristics**			
Female	58 (72.5%)	58 (72.5%)	116 (72.5%)
Age	31.27 (10.76)	32.50 (13.06)	31.89 (11.95)
Married/in a relationship	60 (75.0%)	62 (77.5%)	122 (76.2%)
Number of biological children	2.45 (1.65)	2.69 (1.62)	2.57 (1.64)

Notes: Number of children under age 17 and highest level of education in household includes the highest number/level reported by both caregivers in the household

### Effects of the FSI-ECD + VP

The predicted effects of the intervention on the outcomes at the post-intervention and 3-month time periods are displayed in Table 2 (the original model results showing the time by intervention interaction coefficients are presented in Appendix Table A3). We found that the FSI-ECD + VP was significantly associated with improved caregiver-child interactions. At post-intervention, caregivers in the FSI-ECD + VP arm had higher OCCI scores compared to the control arm (Difference = 3.51, 95% CI: [0.636 6.386], *P* = .017), and higher HOME scores compared to the control arm (Difference = 3.08, 95% CI: 0.889–5.273, *P* = .006). In particular, the intervention arm had significantly higher scores on the HOME “responsiveness” subscale (Difference = 1.66 95% CI: [0.729 2.59], *P* < .001), as well as higher scores on the HOME “organization” subscale (Difference = 0.448 95% CI: [-0.160 1.056], *P* = .149) (Appendix Table A4). There was also some evidence that the FSI-ECD + VP was associated with a lower probability of caregivers experiencing intimate partner-related physical violence at post-intervention (Difference=-0.095, 95% CI: [-0.192 0.002], *P* = .055). We found no statistically significant effect of the intervention on emotion regulation (DERS), post-traumatic stress symptoms or functional impairment (WHODAS). There is some evidence that the FSI-ECD + VP was also associated with fewer symptoms of depression/anxiety at 3-month time point as measured by the HSCL (Difference=-0.191, 95% CI: [-0.374 .-008], *P* = .041), with declines in both the HSCL anxiety subscale (Difference=-0.177, 95% CI: [-0.370 0.016], *P* = .073) and the HSCL depression subscale (Difference=-0.201, 95% CI [-0.396 − 0.005], *P* = .044). We found very similar results when we conducted analyses using imputed data (Appendix Table A5) and when we conducted analyses that included the six replacement households using either complete case analysis (Appendix Table A6) or multiple imputation (Appendix Table A7).

**Table 2:** Model-based predicted effects of the intervention at each time point (*N* = 80 HH, 160 caregivers)

Outcome	FSI Effect: Baseline			FSI Effect: Post			FSI Effect 3 months			Obs
	Coef.	SE	*p*-value	Coef.	SE	*p*-value	Coef.	SE	*p*-value	
WHODAS	-0.60	1.09	0.582	-0.35	1.20	0.769	-1.70	1.20	0.156	418
OCCI	1.09	1.34	0.414	3.51	1.47	0.017	1.27	1.50	0.396	418
DERS	0.64	2.21	0.773	0.90	2.35	0.702	-2.66	2.40	0.268	416
HSCL	0.04	0.09	0.613	0.02	0.09	0.834	-0.19	0.09	0.041	416
HSCL anxiety	0.07	0.09	0.452	0.07	0.10	0.457	-0.18	0.10	0.073	424
HSCL depression	0.05	0.09	0.598	0.00	0.10	0.989	-0.20	0.10	0.044	422
PTSD	-0.19	0.70	0.787	0.33	0.75	0.656	-1.01	0.76	0.185	412
HOME	0.73	1.06	0.491	3.08	1.12	0.006	1.02	1.12	0.363	211
	Prob diff	SE	*p*-value	Prob. Diff	SE	*p*-value	Prob. Diff	SE	*p*-value	Obs
Experienced physical violence in past year	0.00	0.07	0.962	-0.10	0.05	0.055	0.06	0.05	0.275	429
Experienced physical/ sexual violence in past year	-0.02	0.07	0.742	-0.08	0.06	0.150	0.06	0.05	0.293	429

Notes: Coefficients are from linear/logistic models with random effects for household (except for the HOME, which only has one observation per household) and for caregiver. Controls include caregiver age, gender, marital status, highest education level in household, number of children < age 17 in household, child age (months), child sex and district (all measured at baseline)

### Qualitative findings on caregiving practices

Caregivers and CHWs reported that the FSI-ECD + VP positively impacted participants’ lives in a variety of areas, including improved health behaviors, nutrition and feeding practices, relationship quality, and problem-solving skills. All four CHWs observed that mothers interacted with their children and played with them more often after participation in FSI-ECD + VP sessions. Caregivers also noted that they spent more time with their children. One mother stated, “*before*,* we [didn’t] have the time to sit with our children and show them anything*,* but since you started coming*,* we have changed. Now if we come from anywhere*,* we will sit with our children and play with them and show them many things.*” Additionally, caregivers reported increased knowledge of important childcare practices, including learning how to make toys for their children to play with, how to keep children safe from dangerous objects like nails and blades, and how to monitor their child’s overall health. CHWs reported corresponding observation of increased knowledge of childcare practices by caregivers. For example, two CHWs noted caregiver reports of taking their children to health clinic visits more frequently. For example, one CHW said, “testimonies of their children being taken to clinical health care is another reward to me”; and two caregivers mentioned that they learned about the importance of taking their children to the hospital when they were sick. Caregivers also stated that they learned about the importance of hygiene and nutrition for their children, including feeding their children a balanced diet and ensuring that they drink clean water.

### Family dynamics and relationships

Two CHWs noted perceived changes in the relationships between family members and a greater sense of “unity” among them. Improved family unity was a common theme reported by caregivers as well. For example, families changed their process for settling family disputes (not arguing publicly, not involving the children) and they “quarreled” less. Another CHW observed more shared domestic responsibility between female and male caregivers. Likewise, one mother observed that her husband was more engaged and spent more time together with the family:

*My husband is a motor bike rider. Before*,* when night reaches*,* he [goes] out. But now we will sit with not just my children*,* but other children in the house. We will sit and talk about past stories and advise the children that when you go to school*,* they should pay attention to what they have been taught*.

A 42-year-old male CHW supervisor also commented that it is unusual within the culture of Sierra Leone for male caregivers to take on domestic responsibilities:

“*Sitting down with men and talking to them to tell them how they should go about helping their wives to raise their [children] is very difficult in this country*,* but the intervention helped us to be able to convince community members and making men to feel like they are part of the family*,* and they are the ones that own responsibility…to be with their wives in making the lives of this family better*”.

Finally, a 32-year-old male CHW with 8 years of experience commented that the intervention positively impacted his own life:

*I’m so satisfied because the intervention transformed me from one level to the other*,* because I was having problems with my wife and my family*,* but through the intervention I was able to learn a lot. I am now a role model in the community*.

### Appropriateness of FSI-ECD + VP

CHWs and caregivers generally reported that FSI-ECD + VP was appropriate for families with young children living in the rural areas of the Makeni region. Caregivers noted that they were happy that they learned new things, they enjoyed the content on health and hygiene, anger management, and food and nutrition, and they were generally happy with the intervention. Two CHWs noted that the content was clear and a good contextual fit, though this varied somewhat across households. For example, one female CHW noted that while some participants stated that they understood the material, when asked to explain it, their response did not convey a clear understanding. The same CHW further noted that some families had difficulty understanding Krio, potentially due to lower literacy levels, which likely contributed to reading and comprehension difficulties. Another CHW supervisor observed that the pictures in FSI-ECD + VP manuals assisted in caregiver comprehension of the content.

### Feasibility of FSI-ECD + VP

Caregivers and CHWs noted several issues with the feasibility of consistent caregiver participation in the FSI-ECD + VP. Three CHWs noted that caregivers had other responsibilities, such as farm work, which they had to balance with participation in FSI-ECD + VP sessions. CHW SUPs reported that CG fasting and other responsibilities interfered with engaging with the intervention. A 42-year-old male supervisor further noted that holding FSI-ECD + VP sessions during the rainy season posed challenges to attendance because caregivers were often away from their homes to seek food, and traveling to households was difficult due to the poor conditions (i.e., roads flooded, severe mud). While most caregivers did not report any difficulties with feasibility, three said that length of sessions and scheduling were an issue. “*Some of us*,* we are not used to sitting for long*,* and the interview is taking a long time*,” explained one father. Another father explained that after a long day of work, participants wanted to rest rather than engage in a lengthy session, but they still prioritized the program: “*Even if we have things that are ahead of us*,* we will leave everything until after this program ends*.”

CHWs and supervisors both noted that the local fuel crisis stemming from the war in Ukraine impacted the feasibility of FSI-ECD + VP session delivery. Specifically, they explained that with the rising price of fuel, it was difficult to afford transportation to some of the villages where participants resided. Supervisors reported that they were able to provide supervision, though they noted some technical issues with reviewing session recordings due to limited wifi access for downloading and also difficulties charging device and communicating via phone due to the frequent power outages (i.e., electricity was turned off for days at a time to conserve fuel).

### Acceptability of FSI-ECD + VP

In terms of the acceptability of the FSI-ECD + VP among CHWs, they reported enjoying feeling appreciated by caregivers and liking the design and language used in session modules. Caregivers reported that they enjoyed working with their CHWs and reported that the CHWs were able to explain concepts in a clear way and answer questions effectively and kindly. For example, one father explained, *“[The] one lady that was teaching us…that lady was so simple*,* and she doesn’t get angry. She will be calm and responds to us nicely and encourages us. The most important thing that I like about my coach is [she is] patient.”* CHWs reported that they perceived satisfaction with the FSI-ECD + VP from caregivers, and caregivers likewise expressed high satisfaction with the intervention. Some caregivers stated that they wished the FSI-ECD + VP could be expanded to other families and/or that they could continue receiving sessions. Three CHWs noted that the response to the intervention was good, and two commented that families expressed appreciation for the intervention. As one male CHW stated, “*Up until now*,* some people call me expressing thank you messages. Most of the time*,* people call and say*,* we wish this program could have continued*.” Another male CHW observed that participants seemed to become more satisfied as the intervention progressed, but there was some “grumbling” in the early sessions. Similarly, one father stated that it was “painful” at first because they were “wasting” time at the beginning of the intervention, but their CHW coach encouraged them to be patient. After a few more sessions, the father said that the family became “settled” and reduced the amount of arguing.

### Mixed methods findings

Qualitative and quantitative results converged on the finding that FSI-ECD + VP was associated with improved caregiver-child interactions. Specifically, quantitative results showed significantly increased caregiver responsiveness and home organization. Qualitatively, this was triangulated by CHWs’ and caregivers’ reports of increased interactions between caregivers and children, making the home safer, taking children to healthcare clinics, and less arguing between couples. Table 3 demonstrates the alignment between quantitative and qualitative findings related to intervention effectiveness.

**Table 3:** Joint display table with interview quotes that explain quantitative results

Quantitative Finding	Illustrative Qualitative Quote
Higher scores on “responsiveness”	“One of the things I saw after series of [sessions], I saw them playing with their children, dance with their children, sing for them. During their time of cooking, they will call their children and begin to call names of items for example, they say this is cassava I am peeling, this a corn.” - Male CHW with ten years of experience “Firstly, I was the type of mother that always like to stay away when my child is playing, but since after this intervention, I will always stay close to my baby is playing and we can even play together. Also, if my baby wants to go inside and collect something, we will go together and collect it.” - Mother
Higher scores on “organization”	“The family strengthen unit has also created an impact in terms of hygiene aspects. Some people never knew leaving cutlass, knife, blades on the floor of the compound is dangerous to their children. With family strengthen intervention, they have known the importance of not leaving these hazards on the floor in order to save the children. They accepted it and it has helped them a lot. It has helped them in cleaning of the environment such as removal of stagnant water, which is a breeding sites for mosquitoes, this has helped them in preventing their children and themselves from malaria. Through this intervention, people are now adhering to environmental sanitation.” - Male CHW with ten years of experience “I have learned so many things during the intervention. For example, like for us always to take our children to the health centers…to clean the environment, washing of the child’s cloths, taking care of the place where they are sleeping, and more.” - Mother
Less likely to experience intimate partner violence	“Some families were not having peace in their houses, there was no unity. Just like I was saying, if there is no peace in the house from the caregiver, the children will not grow as expected. But when we go and share the ideas with them to tell them that this are [is] the reason that is affecting [their] homes by also showing them a picture to tell them that this is the way you should living [live] as a family so that at the end of the day your children will follow your footsteps. The changes I saw from the families were a [surprise] for me.” – Female CHW with five years of experience “We used to have so many problems…but now I’m seeing changes. We are now in peace.” - Father

With respect to the FSI-ECD + VP’s acceptability and appropriateness among CHWs, quantitative findings indicated that all CHWs felt good about providing the FSI-ECD + VP and thought that the FSI-ECD + VP’s components made ‘a lot of sense’ (Appendix Figure A1). However, some CHWs said that session material may not be entirely clear in the local written language, which was helped by the inclusion of pictures in intervention materials. All 16 CHWs endorsed the FSI-ECD + VP as being helpful for families to raise their children well and as an effective tool to address caregiver mental health and to help families address problems—findings which were consistent across qualitative interviews and quantitative assessments.

Some of the more challenging aspects of implementation were related to resources and timing, with two CHWs reporting that they were dissatisfied with scheduling (i.e., they would have to wait for participants at times, or sometimes they would get to a participant’s house and the participant would say they were not feeling well. They also reported dissatisfaction with their stipend given the increased transportation costs. These findings were consistent across quantitative and qualitative arms of the study. Quantitative survey findings highlighted additional areas where CHWs were less satisfied—including support for self-care and the lack of opportunities for continued clinical support and training.

## Discussion

This study investigated the feasibility, acceptability, and preliminary effects of a culturally adapted version of the FSI-ECD + VP delivered by CHWs to vulnerable families in rural Sierra Leone. Results of qualitative and quantitative analyses converged; findings suggest that caregivers who participated in the FSI-ECD + VP found the intervention acceptable and appropriate, and CHWs found it acceptable and feasible to deliver. Though results should be interpreted with caution, preliminary findings also suggest that caregivers who participated in the FSI-ECD + VP may experience greater improvements in their interactions with their children, and some may experience improved mental health and reduced household violence compared with caregivers who received standard services. Findings are mostly in alignment with prior research on the FSI-ECD + VP (*Sugira Muryango*) in Rwanda [38, 39].

Preliminary quantitative findings suggested that caregivers perceived improvements in their interactions with their children following participation in the FSI-ECD + VP. Qualitative findings also suggested that caregivers who participated in the FSI-ECD + VP perceived an increase in their knowledge and use of positive caregiving practices, and they observed increased engagement in family responsibilities by fathers/male partners. This preliminary data supporting increased knowledge, use of positive caregiving practices, and engagement in family responsibilities by fathers/male partners is worth noting, given the traditional gender norms prevalent in Sierra Leonean society in which childcare is primarily viewed as a woman’s responsibility [40]. However, findings should be interpreted with caution given the smaller sample size of male caregivers and require further examination in a larger, fully powered trial with a longer-term follow-up to better assess the sustainability of potential benefits.

Findings also provide some initial support for the potential of FSI-ECD + VP participation to reduce household violence as well as experiences of depression and anxiety among caregivers. Intervention content focused on developing stress management skills, conflict resolution skills, alternatives to harsh discipline, and increasing father engagement may have contributed to these improvements. For example, it is possible that the decrease in reported household violence may be partially related to positive engagement of male caregivers fostered by the FSI-ECD + VP, as prior studies have suggested a link between male involvement in childcare and reduced intimate partner violence [41, 42]. Sharing caregiving responsibilities can also alleviate burden and stress on female caregivers, which may have positive effects on mental health for both caregivers and their children [43]. Further research is needed to investigate these hypotheses given the small sample size (particularly for male caregivers), lack of power to detect mediators of change, and limited follow-up period. Additionally, these preliminary effects of FSI-ECD + VP participation were marginally significant at 3-month follow-up (though the trends were still towards greater improvements among intervention families). Future implementation efforts might consider adding a booster session after families complete the FSI-ECD + VP and also explore potential moderators of effects (i.e., for whom intervention effects are not sustained).

Findings provide some preliminary support for the feasibility and acceptability of the FSI-ECD + VP among both caregivers and CHWs and highlight several important barriers to the feasibility and acceptability of FSI-ECD + VP delivery by CHWs. The most notable challenges were related to the fuel crisis and the time of year in which the intervention was delivered, both of which made traveling to households very difficult. CHW participants may have become more dissatisfied with FSI-ECD + VP session delivery due to the increased financial burden of transportation costs, difficulties scheduling sessions with families, and difficulties traveling to villages during the rainy season. Future implementation efforts in Sierra Leone should consider how to offset potential financial burdens of transportation (i.e., higher stipends, CHWs only provide home visits to households in greater proximity to their own homes, CHWs from small, hard to reach villages are recruited and trained to serve their village) as well as ensure session delivery is planned according to the season. Despite these challenges, the generally high level of satisfaction with the FSI-ECD + VP reported by both CHWs and caregivers and the perceived cultural relevance and fit may be leveraged to help support future FSI-ECD + VP implementation. It could also be useful to better understand the quality of the relationship between CHWs and caregivers and whether the quality of relationship might influence the intervention’s acceptability and potential benefits.

While study findings are promising in many ways, they should be interpreted considering several limitations. The relatively small sample size limits the generalizability of the findings, and since the study was conducted in a single country, the findings may not be applicable to other contexts. In addition, the study did not include a long-term follow-up, so the sustainability of intervention benefits as well any potential effects on early child development outcomes require further research in a fully powered implementation-effectiveness trial. It is also important to note that the study was a pilot feasibility study–the primary purpose of the study was to assess the feasibility and acceptability of the intervention and to obtain preliminary data on the potential benefits of the FSI-ECD + VP for caregiver-child interactions, caregiver mental health, and violence prevention, not to test the intervention’s effectiveness. Pacing of delivery was impacted by the scheduling challenges, so some families experienced several weeks without receiving a session while others received sessions on a regular weekly basis. The 6-month gap between baseline assessment and intervention delivery may have impacted findings. It is unclear, with the small sample size, whether this uneven delivery schedule may have impacted the ultimate benefit of the intervention among families, or whether it may have helped maintain a longer-term engagement in the ECD sessions.

Additionally, variations in local dialects across the rural region of Makeni may have limited the benefit of the FSI-ECD + VP for those families who had difficulty understanding Krio and received sessions from CHWs who were not fluent in the local dialect spoken by some caregivers. Future research might add more visuals, icons, or other imagery to convey content and/or offer translations of the FSI-ECD + VP in other dialects (i.e., Mende). Finally, we focused on dual caregiver households in the current study. While the criteria for the secondary caregiver was quite flexible, focusing only on dual caregiver households neglects the often-greater needs of single caregiver households in terms of home visiting programs to promote caregiver mental health and ECD outcomes. In Rwanda, the FSI-ECD + VP (*Sugira Muryango*) has been further modified to meet the needs and circumstances of single caregiver families [38, 39]. Future research in Sierra Leone should include these adaptations to the FSI-ECD + VP as a strategy to reach families in even greater need. Future research might also explore strategies to reach those families who are not registered at a PHU or other community clinic (and thus likely have not vaccinated their children or attended any routine visits), as these families may be at even greater risk for household violence, poor caregiver mental health, and poor ECD outcomes.

## Conclusion

In conclusion, this study’s findings suggest that the FSI-ECD + VP, adapted for the Sierra Leonean context, may be feasible and acceptable among families in rural areas and holds promise for promoting positive changes in caregiver-child interactions, caregiver mental health, and the reduction of household violence. Providing additional support for CHW delivered interventions through strategies like transport stipends and ongoing training/supervision could help further increase the feasibility and acceptability of task-sharing approaches in rural and resource-constrained settings. Findings also support the possibility that the FSI-ECD + VP can engage male caregivers in positive parenting practices—a finding that warrants further exploration with a larger sample of male caregivers due to its potential to contribute to broader benefits. Future research should investigate the FSI-ECD + VP’s effectiveness in Sierra Leone via a fully powered implementation-effectiveness trial and continue to cultivate government partnerships to improve the sustainability of task-sharing approaches for evidence-based home visiting interventions.

## Electronic supplementary material

Below is the link to the electronic supplementary material.

Supplementary Material 1

Supplementary Material 2

Supplementary Material 3

## Data Availability

Data will be made available upon reasonable request. All study has been submitted to the NIMH Data Archive and is publicly available at www.nda.nih.gov.

## Abbreviations

CHW: Community Health Worker
DERS: Difficulties in Emotion Regulation Scale
FSI-ECD-VP: Family Strengthening Intervention for Early Childhood Development and Violence-Prevention
HH: Household
HOME: Home Observation for Measurement of the Environment
HSCL: Hopkins Symptom Checklist
IPV: Intimate Partner Violence
LMIC: Low-and Middle-Income Country
OCCI: Observation of Caregiver-Child Interactions
OMIC: Observation of Mother-Child Interactions
RA: Research Assistant
WHODAS: World Health Organization Disability Assessment Schedule
