# Supplementary material for: Task-sharing to promote caregiver mental health, positive parenting practices, and violence prevention in vulnerable families in Sierra Leone: a pilot feasibility study
Source: BMC Psychiatry. 2024 Nov 11;24:787. doi: 10.1186/s12888-024-06209-w (PMC11555851; doi:10.1186/s12888-024-06209-w)
Supplement: Supplementary file 2 — Supplementary Material 2 [file 12888_2024_6209_MOESM2_ESM.docx]

**Study Protocol:**

1. **Study title**: Task-Sharing to Promote Caregiver Mental Health, Positive Parenting Practices, and Violence Prevention in Vulnerable Families in Sierra Leone
2. **Funding**: National Institute of Mental Health (R21 MH124071).

**Research Summary**

- 1. **Introduction and background:**

State the problem and hypothesis

Exposure to war, trauma, and other humanitarian crises can have persistent mental health effects across generations. Our prior study on the intergenerational impact of war in post-conflict Sierra Leone showed that past trauma and exposure to war is related to poor emotion regulation and harsh parenting practices, which adversely affect child development. In prior research among families facing adversity in Rwanda, we developed and evaluated the Family Strengthening Intervention for Early Childhood Development (FSI-ECD/*Sugira Muryango*), a home-visiting behavioral intervention delivered by lay workers. The FSI-ECD targets parental emotion regulation and parent-child interactions as major mechanisms to prevent the intergenerational transmission of emotional and behavioral difficulties related to past trauma. It has demonstrated effectiveness in improving parental emotion regulation, reducing family violence, and promoting healthy child development. The FSI-ECD is a promising approach for targeting underlying mechanisms linked to poor child outcomes. Vital for low-resource settings, it can be delivered feasibly by lay workers with strong supervision. Given the limited health infrastructure in many Low and Middle Income Countries (LMICs), behavioral interventions that can be delivered by well-trained and supervised lay workers are a more viable option for implementation and sustainment of evidence-based practices.

New leadership within the Government of Sierra Leone (GoSL) is focused on mental health initiatives to respond to the Nation’s history of compound adversity and is pursuing mHealth strategies as means to address significant healthcare workforce limitations that plague delivery of evidence-based behavioral interventions to vulnerable families. Mobile technology has the potential to transform health care delivery and improve health outcomes in Sierra Leone and other LMICs by providing training, supervision, and fidelity supports, but it has not been widely applied to mental health and family-based prevention, particularly in Sub-Saharan Africa

Our proposed exploratory research will pilot a culturally adapted version of FSI-ECD delivered by Community Health Workers (CHWs) in Sierra Leone’s underserved, rural Western region. Our project will help build urgently needed capacity both for delivery of evidence-based mental health services to reduce family violence and harsh parenting practices, which can help promote healthy early childhood development.

We will conduct a randomized controlled pilot study to assess feasibility, acceptability, and preliminary effects of mHealth-supported delivery of FSI-ECD on parent mental health, emotion regulation, and familial violence in high risk families with children aged 6–36 months (n=40) in comparison to control families (n=40) who receive standard care. **We hypothesize** (a) the FSI-ECD will be feasible and acceptable among both CHWs and caregivers (b) families who receive the FSI-ECD will show greater improvements in mental health, positive parenting practices, and positive caregiver-child interactions as well as reduced household violence compared with families who receive standard services.

- - 1. Provide the scientific or scholarly reason for this study and background on the topic

**Reducing family violence and harsh parenting practices is a global concern and urgently needed.** War affected populations face higher levels of intergenerational violence. The World Health Organization (WHO) estimates that 35% of women globally report experiencing intimate partner violence in their lifetime, and 75% of children in LMICs experience some form of violent or psychologically damaging discipline at home. Experiencing or witnessing family violence during early childhood increases risks for emotion regulation and other psychological problems, including post-traumatic stress disorder, externalizing and internalizing behavioral difficulties and school problems. In post-conflict Sierra Leone, our prior study on the intergenerational impact of war (R01HD073349) showed that exposure to war violence is related to poor parental mental health and harsh parenting practices. The 2017 Sierra Leone Multiple Indicator Cluster Survey found that 85% of children aged 3-4 and 67% of those aged 1-2 experience violent discipline.^[[1]](#endnote-1)^ These abuses can be fatal; the WHO estimates 41,000 global annual homicide deaths in children under age 15.

**The Family Strengthening Intervention is an evidence-based intervention developed for families facing compounded adversity and has a key focus on promoting parental emotion regulation and preventing family violence.** The Family Strengthening Intervention for Early Childhood Development (FSI-ECD) comprises 5 core components delivered in 12 modules via in-home coaching by CHWs. We developed and tested the FSI-ECD in Sub-Saharan Africa both for families affected by HIV and for families living in extreme poverty. The FSI-ECD focuses on improving transdiagnostic factors (i.e., emotion regulation) to reduce family violence and improve child development outcomes. The FSI-ECD has demonstrated effectiveness in reducing violent child discipline and intimate partner violence and improving parental mental health and functioning. Given that poor parental emotion regulation is related to family violence and poor child development outcomes, the FSI-ECD’s focus on adult emotion regulation and alternatives to violent discipline is a central mechanism of interest related to the intergenerational trauma and violence.

Intervention delivery strategies must innovate to address critical shortages in the mental health workforce in Low and Middle Income Country (LMIC) settings. Access to any form of health service in LMICs is often limited, particularly in rural areas. Sierra Leone’s health infrastructure has been unable to respond effectively to the mental health needs of parents whose lives were affected by war and other forms of adversity. Sierra Leone’s population of nearly 7 million is served by two psychiatrists, fewer than 20 psychiatric nurses, and a handful of doctoral-level mental health professionals. In 2017, the Government of Sierra Leone’s (GoSL) Ministry of Health and Sanitation (MoHS) launched the Community Health Worker Policy, which defines a key role for CHWs in strengthening the delivery of preventative maternal and child health services and linking communities with the health system.

- 1. Specific aims/study objectives
     1. List the purpose of the study

We will pilot a culturally adapted Sierra Leonean version of the FSI-ECD to be delivered by CHWs to vulnerable families with at least one child aged 6-36 months old residing in rural areas of the Makeni region of Sierra Leone.

**Specific Aims:**

**Aim 1.** Conduct a randomized controlled pilot study to assess feasibility, acceptability, and preliminary effects of mHealth-supported delivery of FSI-ECD on parent mental health, emotion regulation, and familial violence in high-risk families with children aged 6–36 months (n=40) in comparison to control families (n=40) who receive standard care.

**Sub-Aim:** We will conduct a cost-effectiveness analysis to assess the economic value of CHW delivery of the FSI-ECD vs. standard services delivered by CHWs.

**Aim 2.** Leverage well-established relationships and GoSL partners to strengthen capacity for mHealth research and quality healthcare delivery in Sierra Leone. We will engage experts in mental health and data science to build in-country capacity in implementation science, mHealth, and behavioral science research. U.S. and Sierra Leonean investigators at the University of Makeni will partner with the GoSL Ministry of Health and Sanitation. Our study will provide a foundation for expanding knowledge, fostering collaboration, and developing research expertise.

- 1. **Materials, methods, and analysis**
     1. **Data collection methods**

We will recruit 40 families to receive the FSI-ECD and 40 control families from rural areas in the Western region of Sierra Leone (N=80 families total; 160 caregivers). To minimize contamination risk, we will use randomization rules developed in our prior work in Sierra Leone (e.g., nonadjacency to reduce potential contact of control and FSI-ECD families). Control families will receive standard maternal and child health services delivered by a CHW via standard supervision. Although this exploratory design does not allow us to parse out the effects of the mHealth supervision tools in comparison to standard FSI-ECD supervision, it does allow us to examine preliminary efficacy of the FSI-ECD with mHealth enhanced supervision and fidelity monitoring. RAs will coordinate participant recruitment via flyers and outreach by local CHWs. All participants will receive a small gift of food.

*Inclusion criteria* are (a) Sierra Leonean household with cohabitating father, mother, and child, with both parents aged 18 or older; (b) having a child aged 6–36 months; (c) one parent scoring at least 62.5 on the DERS. Both parents must agree to attend FSI-ECD sessions. Exclusion criteria are active family crises, i.e., current suicidality or psychosis, serious medical condition, ongoing divorce process.

RAs, trained and supervised by Drs. Desrosiers and Esliker, will screen families for eligibility using a tablet-based survey app to seek informed consent and administer screening measures. This app incorporates algorithms to identify families who meet inclusion criteria and has been used successfully in the ongoing Youth Forward study

*FSI-ECD quantitative data collection* will be at 3 time points: baseline, post-intervention, and 6-month follow-up by UNIMAK RAs. FSI-ECD quantitative outcomes will be parent/caregiver emotion regulation, use of harsh parenting techniques, and family functioning. All measures have been forward and backward translated from English to Sierra Leonean Krio utilizing the WHO process of translation and adaptation. Although the pilot study is not powered to detect treatment effects, we will investigate whether change in core outcomes occurs at post-intervention and 6-month follow-up.

*FSI-ECD qualitative data collection* will be post-intervention via key informant interviews with randomly selected parents (4 males/4 females) to assess feasibility, acceptability, and satisfaction of the FSI-ECD.

- - 1. **Specific materials or tools that will be used to collect the data**

Quantitative assessments will be administered via tablets using the RedCap platform, which is HIPAA compliant. All quantitative measures have been used by the RPCA lab in previous longitudinal research and RCT s in Sierra Leone. From our previous use of these measures with war-affected and/or vulnerable populations in Sierra Leone, Cronbach’s alphas have ranged from .72-.96, demonstrating strong reliability of these measures.

The following measures will be used to assess quantitative mental health outcomes for parents and parent-child interactions.

- Difficulties in Emotion Regulation (DERS) [α=.96]
- WHO Disability Assessment Schedule (WHODAS) [α=.91]
- Post-traumatic Stress Disorder (PTSD) Civilian Checklist [α=.93]
- Demographic and Health Survey IPV items [α=.72-.86]
- Home Observation for Measurement of the Environment (HOME) [α=.73]
- Observation of Mother-Child Interaction (OMCI) [α=.83]
- Hopkins Symptom Checklist [α=.92]

Implementation outcomes will be measured using the following:

- Feasibility, Acceptability, Adoption and Appropriateness will be measured with quantitative scales developed by researchers at Johns Hopkins Bloomberg School of Health (Haroz et al., 2019). We have used these measures in our prior research in Sierra Leone, and the scales demonstrated strong psychometric properties (α= .79 -.90).

Qualitative data will be collected by the University of Makeni RAs, supervised by Dr. Esliker and the in-country Program Manager. For qualitative data collected related to FSI-ECD implementation, RAs will use a semi-structured qualitative interview guide. Family-level key informant interviews on FSI-ECD feasibility and acceptability will be held at participant homes. CHW- and supervisor-level key informant interviews will be held at identified community centers in the target communities Qualitative data in will be audio-recorded, transcribed and translated

For cost-effectiveness data collection, we will use a costing tool developed to collect necessary data. We will use budget, expenditure, supervision, and fidelity data to collect implementation, health, and service costs using standard costing methodologies. Costs will include implementation activities (e.g., staff and CHW/supervisor trainings, session delivery, supervision) and directly related recurrent or capital items (e.g., tablets, tech support, broadband access, travel supplies). Costs of digital tools will be included as a capital item and amortized based on project duration. We will use standard implementation costing techniques where time invested by CHWs and other staff are multiplied by standard salaries plus benefits. Service delivery costs will rely on in-country data or standard costs provided by WHO-CHOICE published costs data. Outcomes will include a functional impairment measure (WHO-DAS) that can be converted to Quality Adjusted Life Year (QALYs). The WHO-DAS will be included in the quantitative assessment battery administered to caregiver participants.

- - 1. **Timeline of the procedures and how long each procedure will last**

**Participation Duration**: *Family participation* is approximately 9 months (weekly FSI-ECD sessions for 3-months and assessments). *CHW/supervisor participation* is approximately 10 months (3-week FSI-ECD training, 1 day technology literacy training, FSI-ECD delivery, and assessments).

- - 1. **Data Analysis Plan**

*Quantitative data analysis* will use mixed effects linear models to assess the effects of the FSI-ECD on caregiver mental health and emotion regulation, parent-child interactions and harsh parenting practices in comparison to standard care (treatment as usual control families). These models will account for clustering of families within CHWs delivering services and clustering of outcomes within families across time. If outcomes are skewed and violate the normality assumption for linear models, we will use generalized linear models with a Poisson distribution. All analyses will be conducted on an intent-to-treat basis. Paired t-tests and Wilcoxon signed rank tests will examine post-intervention change in quantitative dissemination and implementation outcomes of feasibility, acceptability, adoption and appropriate ratings among CHWs and supervisors, and families controlling for baseline scores.

The proposed pilot study is not powered to detect treatment effects of clinical significance. However, if we assume a standard alpha level of 0.05, 80 families with two eligible respondents per family on average, and two time points, with assumptions of moderate intra-class (within-family) correlation (approximately 0.5), this pilot RCT has power of 0.80 to detect a standardized “medium” effect size of approximately 0.50 (Cohen, 1988). For outcomes for which there is only one observation per time point, and using the same assumptions as above, this pilot RCT has power of 0.80 to detect a standardized effect size of approximately 0.6.

Families lost to follow up will be included in all analyses, and depending on the analytic technique, missing time points may be addressed by the method itself (e.g., multilevel modeling), multiple imputation, and/or inverse probability weighting for attrition. All findings will be viewed in the context of lack of power to find effect sizes of clinical significance.

*Qualitative data analysis* of key informant interviews and audio-recorded session content will follow a 3-step analytical strategy derived from thematic content analysis and grounded theory. We will use open-coding to examine key interview themes (e.g., CHW and supervisor experiences with digital interface, barriers and facilitators to use, overall feasibility and acceptability of mHealth tools). We will develop a code book for qualitative analyses of CHWs and supervisors and cross-cutting themes triangulated across data source. We will iteratively develop a coding scheme organized by key themes. Drs. Desrosiers and Betancourt will instruct Dr. Esliker and UNIMAK RAs on use of co-developed code books to independently code 10-20% of transcripts at the appropriate level of analysis to examine reliability in application. Poor agreement (i.e., low kappa ratings as scored in MAXQDA) will be grounds for refining the code book. We will repeat reliability testing until coding is at >80% agreement for all data sources. Once coding operates at high reliability, we will code all datasets in MAXQDA. Mixed methods analysis will synthesize qualitative and quantitative data. This approach will also be used for qualitative data analysis of key informant interviews with parents.

*Cost-effectiveness analysis* will estimate costs across FSI-ECD vs. standard care. We will use budget, expenditure, supervision, and fidelity data to collect implementation, health, and service costs using standard costing methodologies. Costs will include implementation activities (e.g., staff and CHW/supervisor trainings, session delivery, supervision) and directly related recurrent or capital items (e.g., tablets, tech support, broadband access, travel supplies). Costs of digital tools will be included as a capital item and amortized based on project duration. We will use standard implementation costing techniques where time invested by CHWs and other staff are multiplied by standard salaries plus benefits. Service delivery costs will rely on in-country data or standard costs provided by WHO-CHOICE published costs data. Outcomes will include a functional impairment measure (WHO-DAS) that can be converted to Quality Adjusted Life Year (QALYs). We will use standard incremental cost effectiveness analysis to compare mHealth supported delivery of the FSI-ECD to standard care and capture marginal variations in costs and effectiveness using incremental cost-effectiveness ratios (ICERs). Differences in intervention cost will be divided by differences in intervention effectiveness to calculate ICER ratios that can be used to understand the cost of the intervention per unit of outcome (cost per QALY). We can compare this to the standard willingness to pay threshold and to alternative programs to determine which programs are relatively more cost effective

- 1. **Research population and recruitment methods**
     1. **Inclusion and exclusion criteria**

*Families*: We will recruit 40 families to receive the FSI-ECD and 40 control families from rural areas in the Western Region.

**- *Inclusion Criteria:*** We will include families who are (a) a Sierra Leonean household with cohabitating father, mother, and child (aged 6-36 months) with both parents aged 18 or older and; (b) one parent scoring at least 62.5 on the Difficulties in Emotion Regulation Scale (DERS). The DERS cut-off score has been used successfully as a risk assessment screening tool in our prior (R01HD073349) and ongoing studies in Sierra Leone (U19MH109989). Sierra Leonean caregivers scoring above this threshold on the DERS have reported significantly higher levels of severe physical punishment with their children as well as intimate partner violence. Both parents must agree to attend FSI-ECD sessions. If enrolled families have more than one child aged 6-36 months, we will include all eligible children as study participants.

***-Exclusion Criteria:*** We will exclude families who do not meet all inclusion criteria and/or who are experiencing active family crises (e.g., current suicidality or psychosis, serious medical condition, ongoing divorce process).

*Community Health Workers*: We will recruit 8 CHWs from Peripheral Health Units in 2 target communities in coordination with the CHW Focal Person charged with overseeing all CHWs in said communities and the CHW District Coordinator of the Western Region. Traditionally, CHW is a volunteer position and there are no educational qualifications or knowledge and skill prerequisites that must be met in order to be hired as a CHW..

***- Inclusion Criteria*:** CHWs who are 18 years or older and who are assigned to the Peripheral Health Unit that provides health services in one of our target communities.

***- Exclusion Criteria*:** Individuals under age 18 cannot be recruited to work as a CHW.

*Supervisors*: We will recruit 2 supervisors from Peripheral Health Units in the target communities in coordination with the CHW Focal Person and the CHW District Coordinator of the Western Region. Supervisors who participate in AIM 1 Focus Group-

**- Inclusion Criteria:** Supervisors are 18 years or older and oversee CHWs providing maternal and child health services

- **Exclusion Criteria**: Individuals under age 18.
  - 1. **Justification for the number, gender, age, or race of target population**

Our target enrollment for families (parents/caregivers with child), CHWs and supervisors is approximately 50 percent male/50 percent female for each participant group; however, this even representation is a certainty for caregivers only. All participants will be Sierra Leoneans because this is the racial/ethnic demographic composition of the population in Sierra Leone. We will not limit inclusion of any group by sex/gender, race, or ethnicity.

In our prior studies of the Family Strengthening Intervention, our male/female distributions have been roughly equal. Our intent is to recruit families that allow for an equal distribution of male and female children. We aim to recruit an equal number of male and female participants; however, the majority of the CHWs and supervisors in Sierra Leone are female. All data analyses will report qualitative and quantitative data disaggregated by sex. We will also examine sex as a biological variable in our mixed methods analyses.

We will enroll children aged 6-36 months as previous iterations of the FSI-ECD have successfully targeted this group. Excluding children over age 36 months ensures we are targeting families with children in the home versus children attending pre-primary programs, which would increase barriers to intervention delivery with the entire family in the home. Excluding children under age 6 months ensures we are targeting families with children who have developed some degree of mobility and an ability to interact with caregivers rather than passively receive care.

The proposed pilot study of 80 families is not powered to detect treatment effects of clinical significance. However if we assume a standard alpha level of 0.05, 80 families with two eligible respondents per family on average, and two time points, with assumptions of moderate intra-class (within-family) correlation (approximately 0.5), this pilot study has power of 0.80 to detect a standardized “medium” effect size of approximately 0.50 (Cohen, 1988). Enrolling more than 80 families is not feasible given the project budget, timeline, and scope.

- - 1. **Recruitment procedures**

The Program Manager and UNIMAK RAs will recruit families in coordination with the CHW Focal Person, who is the Ministry of Health and Sanitation Community Health Worker Program official responsible for coordinating the work of CHWs and supervisors within peripheral health units. Peripheral Health Units are key units within the Sierra Leone healthcare system. Through smaller community-based subunits, they deliver “first line” care, including prenatal care, routine deliveries, immediate postnatal and neonatal care, community outreach services, routine vaccination, and treatment of childhood illnesses and malnutrition. Peripheral Health Units maintain records of families in the community who have sought services and will be able to identify families with a child aged 6-36 months by reviewing their records. The Program Manager and one UNIMAK RA will conduct screening and consent with families and will obtain informed consent prior to screening. Drs. Esliker and Desrosiers will review the protocol for screening and consent with the Program Manager prior to launching recruitment. The DERS will be used as the eligibility screening tool. The DERS is a 36-item scale that measures difficulties managing emotions (e.g., impulse control). High scores on the DERS have been related to frequency of intimate partner violence (e.g., Gratz & Roemer, 2004). If one parent scores at least 62.5 on the DERS, the family will be eligible. The score of 62.5 is a clinical cut-off that corresponds with a greater likelihood of experiencing clinical disorders (e.g., Staples & Mohlman, 2014). We have used the DERS clinical cut-off successfully as a risk assessment screening tool in our prior (R01HD073349) and ongoing studies in Sierra Leone (U19MH109989). In our prior research, Sierra Leonean caregivers scoring above this threshold on the DERS reported significantly higher levels of severe physical punishment with their children and intimate partner violence. The DERS screener will be administered via tablet, which will automatically calculate a score to determine caregiver eligibility. If eligible, caregivers will be invited to participate in the study and will be asked to provide detailed contact information for future follow-up.

The Program Manager, with the assistance of the Focal Person affiliated with the Peripheral Health Unit in the target community, will recruit CHWs. Equal consideration will be given to all qualified and interested individuals. CHWs will be consented prior to the start of training. CHWs will receive financial support for the duration of their study participation (approximately 9 months) and build skills and capacity in mHealth technology, mental health diagnostics, and intervention delivery with fidelity. We believe that these pieces will a provide strong incentive for CHWs to participate for the duration of the study.

- - 1. **Tools that will be used to recruit**

The Program Manager and UNIMAK RAs will work with the Ministry of Health and Sanitation and the CHW focal person to identify potential participants. UNIMAK RAs will also generate an advertisement/flyer than can be displayed at the Peripheral Health Unit. A recruitment script will be used to explain the study to eligible participants and determine if they are interested in participating. The RAs will attempt to contact potential participants by phone up to three times before traveling to the address provided to contact them in person. The team will attempt three times to contact the potential participants in-person before considering the youth ineligible or uninterested. For those participants who consent to the study, their contact information will be securely stored, and they will be contacted by UNIMAK RAs.

- - 1. **Research incentives and payments**

All participants will be compensated for their participation to include a small household gift of foodstuffs (e.g., soap) equal to 30,000 Leones (equivalent to two days wages in Sierra Leone). As advised by Sierra Leonean researchers and partners at the Ministry of Health and Sanitation, the household gift will be given in lieu of cash in order to maintain a non-coercive, culturally sensitive enrollment process while still providing strong incentives for participation. These incentives will be provided at each time point to encourage participation over time. A participant log will be maintained which will document the participant's unique ID, the time points in which they are participating in either an assessment or interview, and a box to mark when they are provided compensation.

- 1. **Informed consent procedure**

Dr. Esliker will lead RA trainings with the Program Manager. UNIMAK RAs, in close coordination with the Program Manager, will obtain participant consent. Caritas-Freetown will house our in-country Program Manager. Caritas has worked with us during our previous randomized controlled trials in Sierra Leone and oversaw the consent process during these trials.

- - 1. **Assessment of participant understanding of informed consent**

The consent letters will be read out loud to participants to ensure illiteracy does not interfere with the consenting process. Participants will be assured that their decisions about participation (yes or no) will in no way affect their relationship with other service facilitators currently in the community. The consent forms will describe all aspects of the study using literacy-appropriate language including procedures for handling data and explain that confidentiality will be maintained unless concerns about the participant warrant reporting, such as suicidality, homicidality or abuse. The consent will describe the purpose of the study, the participant's involvement, where the study will be conducted, how much time participation is expected to entail, and the information they will be asked to provide. Participants will also be told that participation is voluntary and they are free to withdraw participation at any time. Sufficient time will be allowed for questions about the consent forms or about the study in general.

Participant comprehension of the study, including study details, risks, and benefits, and voluntary participation will be assessed and documented. Drs. Desrosiers, Betancourt, and Esliker will ensure that all research staff are fully trained to conduct the consent process and assess participant comprehension. RAs will evaluate comprehension by asking participants to repeat back their understanding of the study, what their participation entails, and the risks and benefits within. RAs will also use open-ended questions to make sure that potential participants understand all aspects of the study.

- - 1. **Procedures for obtaining informed consent**

RAs will read aloud in Krio, the local language. Reading informed consent forms aloud in Krio is necessary due to the low literacy level in the catchment area for the study. The form will include a description and purpose of the relevant sub-study, procedures to be followed, and what is expected of the participant (e.g., response to questionnaires, protection of confidentiality, the voluntary nature of participation in the intervention and study). In addition, study personnel will discuss any risks related to participation in the study as well as the benefits of participation with the prospective participant.

Sufficient time will be allowed for questions about the consent forms or about the study in general. For participants who request time to consider participation or to discuss the study with family/friends before deciding on participation, they will be given a week to do so. Our research team will follow up with those participants at the end of the one-week period.

Participants will provide verbal consent, which will be logged by the research staff obtaining consent. A research team member will witness the consent and sign the form. Each participant will be offered a copy for his/her reference; the form will include contact information for our Program Manager. Research staff will encourage participants to contact the Program Manager should they have any questions or concerns about the study. Prospective participants also will receive contact instructions for the PI (Dr. Desrosiers), the Sierra Leone Ethics and Scientific Review Committee chair, and the Boston College School of Social Work IRB. If an invited participant declines to participate, we will offer to answer any questions about the study. Only those who consent to participate will be enrolled.

Consent forms will provide participants information regarding conditional assurance of confidentiality for their participation. The section on consent forms indicates the steps the research team will take if they have reason to believe the participant or others are at serious risk for self-harm or harm by others (e.g., current suicidal ideation, reports of physical or sexual abuse). If confidentiality must be breached for a participant, research staff will inform the Project Manager and the PI. Subjects refusing to consent/assent will be thanked for their time and withdrawn from participation.

- 1. **Confidentiality:**
     1. **Data storage and access**

Data will be collected on electronic tablets due to the risk associated with handling paper-based surveys. Tablet collected survey data will be de-identified, stored on encrypted devices, and downloaded routinely on encrypted and password-protected laptops. Trained, supervised research assistants will collect the data and maintain oversight of the password protected tablets. To address concerns of breach of confidentiality, data will be entered into RedCap, an electronic data management software with both online and offline features. Study staff will only able to access RedCap using a secure password on a password-protected tablet. All study documents and data will be maintained in password-protected computer files. All tablets will be kept in secure cabinets with a locking mechanism. Qualitative data collected will be stored electronically, with translations and transcriptions typed to ensure no loss of data. Participant identifiers will be removed from translations and transcriptions. Confidentiality of study documents will be maintained by assigning unique study IDs and using these rather than participant names on all study related materials. Paper copies of documents will be maintained in locked file cabinets in respective countries, which only study staff will have access to. Participant consent forms and ID logs will be kept in a separate location in two separate locked cabinets. Audio recordings will be kept in locked file cabinets and erased at study completion.

- - 1. **Data storage and protection plan**

All study documents and data will be maintained in password-protected computer files or on password protected tablets. Audiotapes and paper copies of documents will be maintained in locked file cabinets and transferred in locked document carrier bags. Participant consent forms and ID logs will be kept in two locked cabinets in a separate location. Transcriptions and audiotapes of interviews will be identified by ID numbers only and not connected with the participants’ names. Source documents will be shredded at the time of study completion in accordance with Boston College Standard Operating Procedures for Researchers Using Human Participants in Research. All hard copies of data will be stored in locked cabinets to which only the local Co-Investigator and RAs have access. After completion of an assessment with a study participant, data with Study ID numbers will be placed in a subject binder in a separate locked file cabinet while waiting for data entry. Once data is entered into computer files and password protected, only the local Co-Investigator, Program Manager, and data entry assistants (RAs) will have access to these files.

- - 1. **Coding and protection of participant identity**

Each district, chiefdom, and participant will have a Study ID#. The Study ID# is a 6-10 digit numerical code depending on the type of participant. It is the link between the participant’s name and the data they provide for the study. A study ID log will be kept separate from study materials in locked cabinets and/or password-protected computers in a secure building at Caritas in Sierra Leone. All documents (e.g. questionnaires) will be labeled with the participant’s Study ID#, not their name. This is to protect the privacy of each participant. All assessment materials will be labeled with the participant’s Study ID#, not their name. This is to protect the privacy of each participant. Study IDs will be pre-populated. The Program Manager and team will work together to prepopulate all the assessment materials prior to data collection.

- 1. **Statement of potential research risks to subjects**

*Planned procedures to protect against data loss or breaches in confidentiality*: Participant diagnostic and assessment data will be collected via tablets by trained UNIMAK RAs. All CHWs will receive training in Good Clinical Practice including proper use of the tablets and transmission of participant data from the tablet to a secure server. During the intervention, CHWs will use the tablets to audiotape sessions. Prior to deployment to the field, the tablets will be encrypted by the Information Technology (IT) department at Boston College. All tablets will be password protected using a password known to the CHW, supervisor, and Program Manager. All data on the tablet will remain on the tablet until it is connected to WIFI and uploaded to a secure server. Daily quality assurance and data monitoring checks will determine successful upload of the data, which will be backed up to Box, a secure, HIPAA-compliant, cloud-based storage platform, before it is remotely wiped from the tablet. In the event a tablet is lost or stolen, Boston College IT can remotely wipe it. We will instruct CHWs that in the event of loss or theft, they must immediately inform their supervisor, who will then immediately notify the Program Manager and PI. The Program Manager will contact Boston College IT with the tablet serial number to initiate a remote wipe of the tablet, The PI will make a report to the Boston College IRB and Sierra Leone Ethics and Research Committee.

*Planned procedures to protect against social harm resulting from breaches in confidentiality*: In the event that a participant reports social harm that results from participation in the study, research study social workers will make every effort to provide appropriate short-term counseling to the subject, and/or referral to appropriate resources for the safety of the participant, as needed. This includes services for housing and food insecurity, domestic violence, mental health, and social services. Information regarding potential social harm will be recorded systematically in study logs. All instances of immediate risk of harm (e.g., situations involving risk of harm to self or others) will activate the study safety plan. This will involve intervention from study social workers and research coordinators, and participation in the study will stop. The PI will also be notified in all such instances. Serious risk of harm cases will be well documented and reported to the IRB, and appropriate referrals will be enacted.

*Planned procedures to protect against distress related to participation in the FSI-ECD*: The FSI-ECD targets vulnerable families with caregivers experiencing impairments in emotion regulation. Based on prior implementation of an adapted version of the FSI-ECD with families in Rwanda experiencing extreme poverty, strong procedures are in place in anticipation of issues that may arise during intervention delivery. A core focus of the FSI-ECD is to improve caregiver-child interaction and the overall functioning of the family. Issues that may cause distress for caregivers include violence against children and intimate partner violence, substance abuse, and caregiver mental health. CHWs will receive extensive training on how to identify these issues when delivering the intervention and how to facilitate conversations with caregivers and support their access of formal supports in the community. Naturalistic videos embedded in the app will provide CHWs with anytime, anywhere access to training and support to help them prepare for these conversations. The CHW supervisor and CARITAS social workers will be an additional source of support and can work with the CHW and family to further address any issues in the home. For cases needing extra support, a trusted community leader may be consulted, and for extreme cases, the risk of harm safety plan may be enacted.

*Planned procedures to protect against distress related to psychosocial assessments*: Some of the content and questions included in the psychosocial assessments may be perceived as sensitive and could cause some level of discomfort for participants. In order to reduce this risk for discomfort, informed consent forms will clearly indicate that individuals unwilling to discuss personal matters may choose not to enroll and may terminate participation at any time. In addition, informed consent forms will emphasize that participants who elect to enroll may choose to abstain from answering any question if it makes them feel uncomfortable.

Adverse events due to participation in psychosocial assessments are anticipated to be rare; however, we have a plan in place to respond should such adverse events occur. We will train the study team in how to identify signs of distress in caregivers. If a participant becomes distressed during the psychosocial assessment, social workers will be available to provide additional individual support and assessment of need for additional mental health services. Should any participant endorse survey items indicating thoughts of suicide, the interviewer will discontinue the survey administration to assess for risk of harm.

In any situation involving study participants in immediate risk of harm (i.e., current suicidal ideation or plan, report of current physical or sexual abuse) the study team member will activate the safety plan.

*Safety plan to identify and address risk of harm cases*: Situations involving study participants in immediate risk of harm will be triaged by the study team member present at the time of interview. The Program Manager and UNIMAK RAs will discuss all risk of harm cases and report them to the PI. The PI will notify the Boston College IRB of risk of harm cases within 24 hours. Risk of harm cases will be referred to appropriate local mental health counselors and emergency facilities as needed within 24 hours. Study social workers or the Program Manager will be available to provide additional individual support and referrals to local social workers for additional mental health services. A standardized form for reporting any activation of the safety plan will be designed and all study team members will be trained in its application. All RAs will complete CITI training on research ethics and compliance and will receive extensive training on the proper protocol for recognizing and responding to risk of harm situations. The following situations will activate the action plan.

*Flagged questions*: Some survey items on the psychosocial assessment are designed to assess participants’ risk behaviors and welfare, including critical issues such as suicidal ideation, physical abuse, and sexual abuse. Specific questions related to these problems are “flagged”; a positive response to flagged questions will mandate activation of the safety plan. The use of flagged questions is intended to minimize variability in research staff response and to ensure that all research personnel respond to certain indicators of risk of harm.

*Concerning scores*: For example, a concerning score for intimate partner violence reported by a participant will activate the safety plan.

Potential action plan responses are:

*If suicidal ideation is reported by a participant*: Research staff will conduct an immediate follow-up assessment with the participant. Staff will use scripts, which have been developed for these follow-up sessions, and include specific text for discussing mental health problems and access to services (e.g., “Based on your responses to some of these questions, I have concerns about your safety and would like to have someone on our team touch base with you”). After having this session with any participants in distress, staff will contact the Program Manager for an initial assessment of suicidal risk and will inform the PI. Trained study social workers from CARITAS will conduct this assessment to determine current suicidal ideation or psychosis. Appropriate referrals will be made to the most suitable mental health services (e.g., social work, psychology, or psychiatry) depending on level of clinical need. Referral services will be contacted if suicidal ideation is ongoing and the participant cannot be kept safe with social work and family level interventions.

*If ongoing abuse is reported by a participant*: Research staff will inform the Program Manager or PI, and if appropriate, a local authority through the current legal, health, and social services channels. If reported abuse relates to a child in the study, a report will be submitted to the Ministry of Social Welfare District Council or Family Support Unit of the Police, as required by national law. In abuse cases involving participants, the safety plan recommends informing the participants’ primary care or mental health counselors.

*If a participant scores at very high levels of intimate partner violence or indicates ongoing intimate partner violence*: Research study social workers will perform an initial evaluation, confer with the Program Manager and PI, and refer for appropriate care through the primary care counselor or mental health provider.

*Referrals for participants in need of stage two treatment*: At the time of psychosocial assessment interviews, participants will be asked questions to assess for anxiety and stress. Participants will be asked, “Do you feel you need any additional care or services due to your experiences today? Are there any questions or concerns that you would like to discuss with a mental health provider or other health professional?” Any risk of harm concerns will be discussed at this time. If needed, the counselors will provide referrals to appropriate services. All questions will be referred to Dr. Desrosiers and the Program Manager. All Safety Plan Activation Forms will be saved as an encrypted file in a password protected folder on a password protected computer managed by the Program Manager. This folder will also be saved on Box, a secure HIPAA-compliant, cloud-based platform. The study team will minimize the use of paper logs, instead utilizing computers and tablets to track participant-related study logs.

We will refer study participants who show persistent impairment and distress due to post-traumatic stress disorder, anxiety, depression, or mental health concerns to mental health nurses with the Peripheral Health Unit in the community or to other community programs. If necessary and with the consent of participants, a trusted community or family member may also be enlisted as a source of interpersonal support. The Program Manager and research staff will discuss all cases requiring treatment and report them to the PI. We will design a standardized form for reporting referrals; all study team members will be trained in its application.

This protection against risk of harm plan has been successfully implemented and used in our prior work in Sierra Leone (U19MH109989/Youth Forward). RAs in the Youth Forward study have conducted resource and referral mapping to ensure that the appropriate referrals can be made given the geographic location of participants. Available health-related services as well as non-government organizations (NGOs) and community-based organizations have been documented at three levels: the community level, the chiefdom level, and the district level. This data provides a map of available referral services from which the flow of referrals can be documented depending on the situation. This ensures referrals are made to clinics and mental health professionals that are accessible to each participant and appropriate to their specific situation.

- - 1. **Likelihood and magnitude of the risks or discomforts occurring**

Participants may experience psychological or privacy risks; however, the likelihood and magnitude of risks or discomforts occurring are anticipated to be low.

- - 1. **Minimization of the risk**

Some of the content and questions included in the psychosocial assessments may be perceived as sensitive and could cause some level of discomfort for participants. We will minimize risks by obtaining voluntary consent from all participants using informed culturally responsive consent procedures consistent with IRB guidelines. In order to reduce this risk for discomfort, informed consent forms will clearly indicate that individuals unwilling to discuss personal matters may choose not to enroll and may terminate participation at any time. In addition, informed consent forms will emphasize that participants who elect to enroll may choose to abstain from answering any question if it makes them feel uncomfortable.

Adverse events and due to participation in psychosocial assessments and key informant interviews are anticipated to be rare; however, we have a plan in place to respond should such adverse events occur. We will train research assistants in how to identify signs of distress in participants. If a participant becomes distressed during the psychosocial assessment, social workers will be available to provide additional individual support and assessment of need for additional mental health services. Should any participant endorse survey items indicating thoughts of suicide, the interviewer will discontinue the survey administration to assess for risk of harm. In any situation involving study participants in immediate risk of harm (i.e., current suicidal ideation or plan; report of current physical or sexual abuse) the research team member will activate the safety plan (see above response for more detailed information on the safety plan).

If a participant decides to withdraw from the study, his/her reasons for withdrawal will be documented. The research team will refer the participant to the appropriate mental health services if needed. Our research team in Sierra Leone maintains a strong referral network to include universities with access to clinical staff in medical school and

university-affiliated hospitals, and will refer to these resources when participants need mental health care. If a participant withdraws, all data collected up to that point will remain and be used in the study, unless otherwise indicated by the participant (which will be documented in the subject log).

All members of the research team will be trained in research ethics, including methods to protect participant confidentiality and the importance of that protection. Special emphasis will be placed on providing training in ethical research conduct for the local Sierra Leonean CWHs, supervisors and research assistants. In particular, for team members who reside in the study catchment area, training will focus on the maintenance of confidentiality when performing research.

We will enroll children aged 6-36 months in the study. Caregivers must provide informed assent for children to participate in assessments and the FSI-ECD. During data collection, if a child presents with an issue necessitating additional care (e.g. malnutrition), the team member will notify the Program Manager and CHW supervisor to make a referral for additional services. Study RAs will be trained in use of an anthropometrics assessment tool to measure upper arm circumference as an indicator of malnutrition. This approach has been used successfully in prior research on the FSI-ECD in Rwanda to indicate malnutrition (NCT02510313). If there is a case of suspected abuse or neglect, the RA will enact the safety plan immediately to ensure that proper services are in place for the child and family.

- 1. **Statement of potential research benefits to subjects**

In prior implementation of the FSI-ECD in Rwanda, participants reported that their study participation helped them to improve their ability to care for their children and work in partnership with their intimate partner and resolve conflicts in healthy ways. Potential benefits for families enrolled in the FSI-ECD include enhanced contact with trained CHWs. A tablet interface that integrates validated diagnostic tools (i.e., the DERS, CTS, HOME) will enable CHWs to identify and monitor family violence and mental health issues and potentially provide the Ministry of Health and Sanitation with surveillance data to inform policy and service delivery decisions**.** CHWs will be able to view results of mHealth data obtained on these diagnostics on dashboards via user-friendly visualization tools (e.g., charts, summaries) to enable immediate identification of families with concerning scores on indicators of violence or mental health problems as well as easier tracking of changes over time. Given the focus of the FSI-ECD, caregivers enrolled in the study may experience improved emotion regulation, with positive impacts on one’s ability to utilize healthy coping strategies and respond to stressful situations. A structured safety plan, which includes a clinical supervisor, CARITAS social workers, and respected community leaders, will be in place to assist families in navigating external formal support to address issues requiring a higher level of care. When these problems are addressed promptly and appropriately, the caregivers and their children will benefit.

Potential benefits for CHWs include the ability to expand their capabilities knowledge and skills in providing evidence-based services for early childhood development. They may also experience an enhanced supervision experience and/or greater support from supervisors through our strong supervision plan. As part of our fidelity monitoring and implementation approach, CHWs will participate in structured supervision that targets CHW capacity and skill building related to intervention delivery. As such, CHWs will receive one-on-one feedback regarding their strengths and weaknesses, with targeted recommendations and direction for improvement. Enhanced supervision for CHWs will not only improve their abilities as CHWs it will improve how the FSI-ECD is delivered in the home, which will strengthen FSI-ECD impact for participating families. Finally, CHWs in our study will be better positioned to meet the national priorities outlined in the National Community Health Worker Policy.

All participants will be engaged in research that may benefit families facing adversity in Sierra Leone and more broadly in Sub-Saharan Africa and other LMICs. Participants may gain emotional benefit or satisfaction from contributing to the body of knowledge regarding health and populations facing compounded adversity. Our data will be used to identify issues of priority to policy makers and program developers that can be the targets of improved policies and services in this and other settings. Beyond Sierra Leone, the general patterns and lessons learned from this research have the potential to improve services and service delivery for vulnerable families and to expand the reach of services to other regions. The data will not only contribute to implementation science research, but also will help our NGO partners, UN agencies, and other policy audiences to understand the nature of this population’s needs and inform decision-making regarding scaling up of the m-health supported FSI-ECD.

**4. Dissemination**

Study leaders are committed to the timely dissemination of research outcomes. The Program Manager will be responsible for handling ClinicalTrials.gov requirements for this project under the PI’s oversight. The Program Manager will work with the PI to register the trial prior to enrolling the first subject. Once a record is established, the Program Manager will confirm accuracy of record content, resolve problems, and maintain records, including content update and modifications. The Program Manager also will be responsible for aggregate results reporting and adverse event/serious adverse event reporting at the conclusion of the project. The data generated in this grant will be presented at national or international conferences and published in a timely fashion. All final peer-reviewed manuscripts that arise from this proposal will be submitted to the digital archive PubMed Central.

1. [↑](#endnote-ref-1)
