# Supplementary material for: Task-sharing to promote caregiver mental health, positive parenting practices, and violence prevention in vulnerable families in Sierra Leone: a pilot feasibility study
Source: BMC Psychiatry. 2024 Nov 11;24:787. doi: 10.1186/s12888-024-06209-w (PMC11555851; doi:10.1186/s12888-024-06209-w)
Supplement: Supplementary file 3 — Supplementary Material 3 [file 12888_2024_6209_MOESM3_ESM.docx]

**Appendix Table A1. Baseline levels of outcomes in study sample**

|  | Control (N=80) | Intervention (N=80) | Total (N=160) |
| --- | --- | --- | --- |
| Outcome measure | Mean (SD) or N (%) | | |
| WHO Disability Assessment Scale (WHODAS 12-item sum score) | 7.59 (6.74) | 7.11 (6.36) | 7.35 (6.54) |
| Observation of Caregiver-Child Interactions (OCCI 19-item sum score) | 28.57 (9.13) | 28.73 (9.21) | 28.65 (9.14) |
| Difficulties in Emotional Regulation (DERS 36-item sum score) | 68.03 (12.33) | 68.71 (16.23) | 68.36 (14.35) |
| Hopkins Symptom Checklist (HSCL-25-item mean score) | 1.77 (0.49) | 1.80 (0.60) | 1.79 (0.55) |
| Hopkins Symptom Checklist Anxiety subscale (10-item mean score) | 1.78 (0.53) | 1.84 (0.62) | 1.81 (0.58) |
| Hopkins Symptom Checklist Depression subscale (15-item mean score) | 1.75 (0.51) | 1.79 (0.65) | 1.77 (0.58) |
| Post-traumatic Stress Symptoms (PTSD 16-item sum score) | 3.99 (3.55) | 3.61 (4.07) | 3.80 (3.81) |
| HOME (Home Observation Measurement of the Environment 43-item sum score) | 23.31 (3.95) | 23.47 (4.15) | 23.39 (4.02) |
| Experienced physical violence in past year | 22 (27.5%) | 23 (28.8%) | 45 (28.1%) |
| Experienced sexual violence in past year | 6 (7.5%) | 5 (6.2%) | 11 (6.9%) |
| Experienced physical or sexual violence in past year | 24 (30.0%) | 23 (28.8%) | 47 (29.4%) |

**Appendix Table A2: Characteristics of intervention households that remained in the study (N=33) compared to those that left the study after the baseline survey (N=7)**

|  | | Intervention HH remained in study (N=33 HH, 66 cargivers) | Intervention HH lost after baseline (N=7HH, 14 caregivers) | |  |
| --- | --- | --- | --- | --- | --- |
|  | N (%) or Mean (SD) | | | | |
| **Household Characteristics** |  | | |  | |
| Location |  | | |  | |
| Yoni | 16 (48.5%) | | | 2 (28.6%) | |
| Binkolo | 17 (51.5%) | | | 5 (71.4%) | |
| Number of children under 17* | 4.85 (2.75) | | | 5.71 (4.39) | |
| Highest level of education in household* |  | | |  | |
| Primary school | 5 (15.2%) | | | 0 (0.0%) | |
| Junior secondary | 7 (21.2%) | | | 1 (14.3%) | |
| Senior secondary | 17 (51.5%) | | | 3 (42.9%) | |
| Post-secondary | 4 (12.1%) | | | 3 (42.9%) | |
| Focal child age (months) | 21.51 (9.59) | | | 13.64 (3.28) | |
| Focal child female | 10 (30.3%) | | | 3 (42.9%) | |
| Primary caregiver relationship with child | | | |  | |
| Mother | 32 (97.0%) | | | 6 (85.7%) | |
| Grandmother | 1 (3.0%) | | | 0 (0.0%) | |
| Other | 0 (0.0%) | | | 1 (14.3%) | |
| Secondary caregiver relationship with child |  | | |  | |
| Father | 19 (57.6%) | | | 2 (28.6%) | |
| Grandmother | 9 (27.3%) | | | 2 (28.6%) | |
| Aunt | 2 (6.1%) | | | 1 (14.3%) | |
| Other | 3 (9.1%) | | | 2 (28.6%) | |
| **Caregiver Characteristics** |  | | |  | |
| Female | 46 (69.7%) | | | 12 (85.7%) | |
| Age | 32.30 (12.35) | | | 33.43 (16.53) | |
| Married/in a relationship | 56 (84.8%) | | | 6 (42.9%) | |
| Number of biological children | 2.82 (1.59) | | | 2.07 (1.69) | |

Notes: Number of children under age 17 and highest level of education in household includes the highest number/level reported by both caregivers in the household. Sample is limited to intervention households only. The only variables that were significantly different between both groups at P<.05 were the child's age and the caregiver's marital status

**Appendix Table A3: Results of mixed models predicting caregiver outcomes (N=80 Households, 160 caregivers)**

|  | FSI Effect Post | | |  | FSI Effect 3 months | | | Number of observations |
| --- | --- | --- | --- | --- | --- | --- | --- | --- |
| Outcome | Coefficient | SE | p-value |  | Coefficient | SE | p-value |  |
| WHODAS | 0.25 | 1.45 | 0.862 |  | -1.09 | 1.45 | 0.449 | 418 |
| OCCI | 2.42 | 1.79 | 0.178 |  | 0.18 | 1.82 | 0.922 | 418 |
| DERS | 0.26 | 3.01 | 0.931 |  | -3.30 | 3.05 | 0.279 | 416 |
| HSCL | -0.02 | 0.11 | 0.830 |  | -0.23 | 0.11 | 0.033 | 416 |
| HSCL anxiety | 0.01 | 0.12 | 0.964 |  | -0.25 | 0.12 | 0.036 | 424 |
| HSCL depression | -0.05 | 0.12 | 0.689 |  | -0.25 | 0.12 | 0.034 | 422 |
| PTSD | 0.52 | 0.90 | 0.561 |  | -0.82 | 0.91 | 0.367 | 412 |
| HOME | 2.35 | 1.35 | 0.082 |  | 0.29 | 1.35 | 0.831 | 211 |
|  | OR | SE | p-value |  | OR | SE | p-value | Number of observations |
| Experienced physical violence in past year | 0.26 | 0.21 | 0.097 |  | 2.03 | 1.50 | 0.341 | 429 |
| Experienced physical/ sexual violence in past year | 0.46 | 0.34 | 0.287 |  | 2.40 | 1.81 | 0.248 | 429 |

Note: Coefficients are from mixed effects linear/logistic models with random effects for household (except for the HOME which only has one observation per household) and for caregiver. Controls include caregiver age, caregiver gender, caregiver marital status at baseline, highest education level in household, number of children under 17 at baseline in household, child age (months), child sex and district. Table reports coefficients on the interaction term between the time period and the intervention group.

**Appendix Table A4: Model-based predicted effect of intervention for OCCI (N=160 caregivers) and HOME (N=80 caregivers) subscales**

|  | FSI Effect: Baseline | | | FSI Effect: Post | | | FSI Effect 3 months | | | Number of obs |
| --- | --- | --- | --- | --- | --- | --- | --- | --- | --- | --- |
| Outcome | Coef | SE | p-value | Coef | SE | p-value | Coef | SE | p-value |  |
| **OCCI** |  |  |  |  |  |  |  |  |  |  |
| OCCI | 1.09 | 1.34 | 0.414 | 3.51 | 1.47 | 0.017 | 1.27 | 1.50 | 0.396 | 418 |
| OCCI- child | 1.33 | 0.70 | 0.057 | 1.84 | 0.77 | 0.016 | 0.77 | 0.78 | 0.324 | 418 |
| OCCI- mother | -0.30 | 0.81 | 0.716 | 1.66 | 0.90 | 0.065 | 0.67 | 0.91 | 0.460 | 421 |
| **HOME** |  |  |  |  |  |  |  |  |  |  |
| HOME | 0.73 | 1.06 | 0.491 | 3.08 | 1.12 | 0.006 | 1.02 | 1.12 | 0.363 | 211 |
| HOME- responsive | 0.37 | 0.44 | 0.396 | 1.66 | 0.47 | 0.000 | 0.11 | 0.47 | 0.822 | 214 |
| HOME- accept | 0.03 | 0.20 | 0.864 | 0.26 | 0.21 | 0.220 | 0.31 | 0.21 | 0.142 | 216 |
| HOME- organization | 0.02 | 0.29 | 0.951 | 0.45 | 0.31 | 0.149 | -0.20 | 0.31 | 0.510 | 214 |
| HOME- learning | 0.23 | 0.42 | 0.591 | 0.18 | 0.46 | 0.704 | 0.24 | 0.46 | 0.604 | 216 |
| HOME- involve | 0.06 | 0.27 | 0.824 | 0.34 | 0.29 | 0.243 | 0.12 | 0.29 | 0.693 | 216 |
| HOME variety | 0.13 | 0.24 | 0.582 | 0.21 | 0.26 | 0.420 | 0.41 | 0.26 | 0.124 | 215 |

Note: Coefficients are predicted differences between intervention and control arms from mixed effects linear/logistic models with random effects for household (except for the HOME scales which only has one observation per household) and for caregiver. Controls include caregiver age, caregiver gender, caregiver marital status at baseline, highest education level in household, number of children under 17 at baseline in household, child age (months), child sex and district.

**Appendix Table A5: Model-based predicted effect of intervention at each time point using multiple imputation (N=80 HH, 160 caregivers)**

|  | FSI Effect: Baseline | | | FSI Effect:Post | | | FSI Effect 3 months | | | Number of obs |
| --- | --- | --- | --- | --- | --- | --- | --- | --- | --- | --- |
| Outcome | Coefficient | SE | p-value | Coefficient | SE | p-value | Coefficient | SE | p-value |  |
| WHODAS total | -0.77 | 1.09 | 0.479 | -0.49 | 1.21 | 0.685 | -1.92 | 1.16 | 0.098 | 480 |
| OMCI total | 1.03 | 1.38 | 0.453 | 4.66 | 1.59 | 0.004 | 0.86 | 1.62 | 0.596 | 480 |
| DERS total | 0.80 | 2.24 | 0.721 | 1.01 | 2.37 | 0.671 | -3.19 | 2.42 | 0.188 | 480 |
| HSCL total | 0.06 | 0.09 | 0.482 | 0.02 | 0.09 | 0.799 | -0.22 | 0.09 | 0.020 | 480 |
| PTSD | -0.17 | 0.70 | 0.815 | 0.31 | 0.77 | 0.683 | -1.12 | 0.79 | 0.155 | 480 |
| HOME | 0.87 | 1.08 | 0.419 | 3.00 | 1.15 | 0.010 | 0.95 | 1.14 | 0.403 | 240 |

Note: Coefficients are predicted differences between intervention and control arms from mixed effects linear/logistic models with random effects for household (except for the HOME which only has one observation per household) and for caregiver. Controls include caregiver age, caregiver gender, caregiver marital status, highest education level in household, number of children under 17 in the household, child age (months), child sex and district (all measured at baseline). Missing data was imputed using multiple imputation with chained equations and 20 multiply imputed datasets. Models with the binary IPV outcomes and the HSCL sub-scales did not converge.

**Appendix Table A6: Model-based predicted effect of intervention at each time point including 6 replacement households (N=86HH, 172 caregivers)**

|  | FSI Effect: Baseline | | | FSI Effect:Post | | | FSI Effect 3 months | | | Number of obs |
| --- | --- | --- | --- | --- | --- | --- | --- | --- | --- | --- |
| Outcome | Coef | SE | p-value | Coef | SE | p-value | Coef | SE | p-value |  |
| WHODAS | -0.58 | 1.09 | 0.596 | -0.57 | 1.15 | 0.618 | -1.13 | 1.14 | 0.321 | 441 |
| OCCI | 0.98 | 1.35 | 0.467 | 3.28 | 1.42 | 0.021 | 0.87 | 1.44 | 0.546 | 441 |
| DERS | 1.04 | 2.22 | 0.639 | 2.04 | 2.26 | 0.367 | -1.23 | 2.30 | 0.592 | 440 |
| HSCL | 0.05 | 0.09 | 0.550 | 0.02 | 0.09 | 0.853 | -0.14 | 0.09 | 0.114 | 439 |
| HSCL anxiety | 0.07 | 0.09 | 0.417 | 0.06 | 0.10 | 0.523 | -0.13 | 0.09 | 0.181 | 448 |
| HSCL depression | 0.06 | 0.09 | 0.545 | -0.00 | 0.10 | 0.975 | -0.15 | 0.10 | 0.113 | 445 |
| PTSD | -0.15 | 0.69 | 0.833 | 0.38 | 0.71 | 0.592 | -0.75 | 0.73 | 0.303 | 435 |
| HOME | 0.77 | 1.05 | 0.460 | 3.09 | 1.06 | 0.004 | 1.25 | 1.06 | 0.240 | 223 |
|  | Prob diff | SE | p-value | Prob. Diff | SE | p-value | Prob. Diff | SE | p-value | Number of obs |
| Experienced physical violence in past year | 0.00 | 0.07 | 0.952 | -0.09 | 0.05 | 0.073 | 0.05 | 0.05 | 0.282 | 453 |
| Experienced physical/ sexual violence in past year | -0.02 | 0.07 | 0.737 | -0.08 | 0.05 | 0.153 | 0.05 | 0.05 | 0.301 | 453 |

Note: Coefficients are predicted differences between intervention and control arms from mixed effects linear/logistic models with random effects for household (except for the HOME which only has one observation per household) and for caregiver. Controls include caregiver age, caregiver gender, caregiver marital status, highest education level in household, number of children under 17 in household, child age (months), child sex and district (all measured at baseline).

**Appendix Table A7: Model-based predicted effect of intervention at each time point using multiple imputation and including 6 replacement households (N=86 HH, 172 caregivers)**

|  | FSI Effect: Baseline | | | FSI Effect:Post | | | FSI Effect 3 months | | | Number of obs |
| --- | --- | --- | --- | --- | --- | --- | --- | --- | --- | --- |
| Outcome | Coef | SE | p-value | Coef | SE | p-value | Coef | SE | p-value |  |
| WHODAS total | -0.69 | 1.08 | 0.523 | -0.75 | 1.12 | 0.500 | -1.16 | 1.12 | 0.300 | 516 |
| OMCI total | 1.19 | 1.39 | 0.391 | 3.81 | 1.51 | 0.012 | 0.67 | 1.54 | 0.666 | 516 |
| DERS total | 0.90 | 2.23 | 0.686 | 2.22 | 2.24 | 0.323 | -1.43 | 2.27 | 0.528 | 516 |
| HSCL total | 0.07 | 0.09 | 0.437 | 0.02 | 0.09 | 0.855 | -0.15 | 0.09 | 0.096 | 516 |
| PTSD | -0.09 | 0.69 | 0.898 | 0.32 | 0.72 | 0.662 | -0.81 | 0.76 | 0.285 | 516 |
| HOME | 1.03 | 1.05 | 0.327 | 3.27 | 1.06 | 0.002 | 1.34 | 1.13 | 0.237 | 258 |

Note: Coefficients are predicted differences between intervention and control arms from mixed effects linear/logistic models with random effects for household (except for the HOME which only has one observation per household) and for caregiver. Controls include caregiver age, caregiver gender, caregiver marital status, highest education level in household, number of children under 17 in household, child age (months), child sex and district (all measured at baseline). Missing data was imputed using multiple imputation with chained equations and 20 multiply imputed datasets. Models with the binary IPV outcomes and HSCL subscales did not converge

**Appendix Figure A1. CHW perceptions of FSI-ECD+VP feasibility, acceptability, and appropriateness**
